# Novel somatic variants involved in biochemical activity of pure growth hormone-secreting pituitary adenoma without *GNAS* variant

**DOI:** 10.1038/s41598-021-95829-3

**Published:** 2021-08-16

**Authors:** Cheol Ryong Ku, Hyeonseob Lim, Yang Jong Lee, Sun Ho Kim, Daham Kim, Se Hoon Kim, Mi Kyung Lee, Duhee Bang, Eun Jig Lee

**Affiliations:** 1 Institute of Endocrine Research, Pituitary Tumor Center, Yonsei University College of Medicine, 50-1 Yonsei-ro, Seodaemun-gu, Seoul, 03722 Korea; 2 Department of Chemistry, Yonsei University, 50 Yonsei-ro, Seodaemun-gu, Seoul, 03722 Korea; 3 Department of Neurosurgery, Yonsei University College of Medicine, Seoul, South Korea; 4 Department of Neurosurgery, Ewha Womans University School of Medicine, Seoul, South Korea; 5 Department of Pathology, Yonsei University College of Medicine, Seoul, South Korea; 6 Department of Pathology, National Health Insurance Service Ilsan Hospital, Gyeonggi-do, Republic of Korea

**Keywords:** Genetics, Neuroscience, Endocrinology

## Abstract

We aimed to identify somatic genetic alterations in pure growth hormone (GH)-secreting pituitary adenomas without *GNAS* variants. Patients with GH-secreting pituitary adenoma who underwent transsphenoidal adenomectomy at Severance Hospital, Yonsei University College of Medicine were recruited. Somatic genetic alterations were profiled by whole-exome sequencing (WES) and targeted resequencing. WES was performed using DNA from nine GH-secreting pituitary tumors and corresponding blood samples. Absence of *GNAS* variant was confirmed by Sanger sequencing. For targeted resequencing of 140 fixed tissues, 48 WES-derived candidate genes and 7 GH-secreting pituitary adenoma-associated genes were included. Forty-eight genes with 59 somatic variants were identified by WES. In targeted resequencing, variants in 26 recurrent genes, including *MAST4, PRIM2*, *TNN*, *STARD9*, *DNAH11*, *DOCK4*, *GPR98*, *BCHE*, *DARS*, *CUBN*, *NGDN*, *PLXND1*, *UNC5B*, and *COL22A1*, were identified, but variants in previously reported genes were not detected. *BCHE*, *DARS*, *NGDN*, and *UNC5B* variants were associated with increased GH-secreting pituitary tumor biochemical activity, which was confirmed in vitro. Although recurrent point variants were rare, several somatic variants were identified in sporadic pure GH-secreting pituitary adenomas. Several somatic variants may affect pathways involved in the tumorigenesis and biochemical activities of GH-secreting pituitary adenomas.

## Introduction

Growth hormone (GH)-secreting pituitary adenoma is the most common cause of acromegaly, which is associated with high mortality caused by cardiovascular disease, metabolic disorders, and malignancies^1–3^. Many pituitary adenomas have a monoclonal origin, indicating that they are formed from the replication of a single cell that gained a growth advantage, possibly as a result of genetic or epigenetic modifications leading to proto-oncogene activation or tumor suppressor gene inactivation^4,5^. Despite extensive research, however, little is known about the genetic causes of pituitary adenomas. Although most pituitary adenomas are sporadic tumors, the only variants found in most cases of sporadic GH-secreting adenomas to date (approximately 35–40%) occur in the gene for the stimulatory G-protein α-subunit (*GNAS*)^6–8^.

Understanding the tumor pathology requires identifying and understanding the genetic alterations that drive tumorigenesis initiation and progression. The advancement of powerful computational methods, combined with next-generation sequencing technology, allows for the characterization of the somatic landscape through human tumors and variants important for diagnostic and therapeutic purposes^9,10^. The somatic landscape has been studied in several sporadic pituitary adenomas by exome sequencing^11–13^. However, few variants, such as the *USP8* variant in Cushing’s disease, have been identified by exome sequencing.

**Figure 1:**
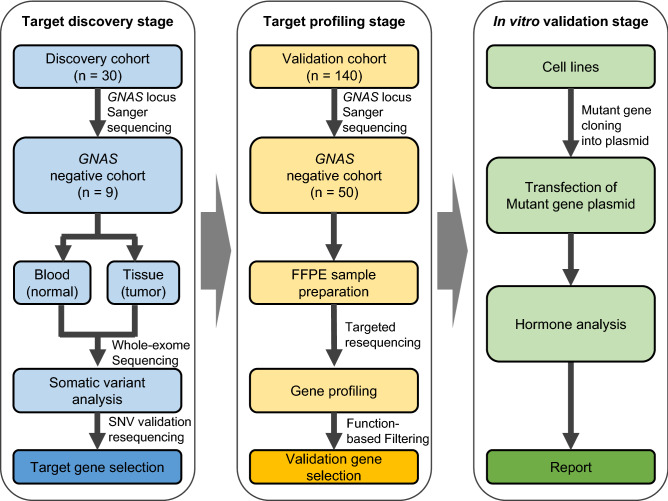
Schematic flow diagram of the current study. Genetic variants were investigated in three stages. In the first stage, “Target Discovery Stage,” blood and tissue samples of 30 patients were analyzed by Sanger sequencing, whole-exome sequencing (WES), and single nucleotide variant (SNV) validation resequencing, and genes with somatic variants were identified as target genes for profiling. In the second stage, “Target Profiling Stage,” target genes from 140 FFPE samples were analyzed by targeted resequencing, and relevant variants were selected for further validation. In the third stage, “In vitro Validation Stage,” selected variants were functionally examined by in vitro validation. Expression vectors containing variants were constructed and transfected into GH3 cell lines (drawn in Microsoft 365 Powerpoint; https://www.microsoft.com/ko-kr/microsoft-365).

We performed somatic variant profiling based on whole-exome sequencing (WES) and targeted resequencing analysis in a large and well-characterized collection of 50 of 140 patients with acromegaly with pure GH-expressing pituitary adenomas without *GNAS* variants (Fig. 1). We aimed to identify the somatic variants that may be associated with clinical differences in patients with GH-secreting pituitary adenoma.

## Results

### WES

**Figure 2:**
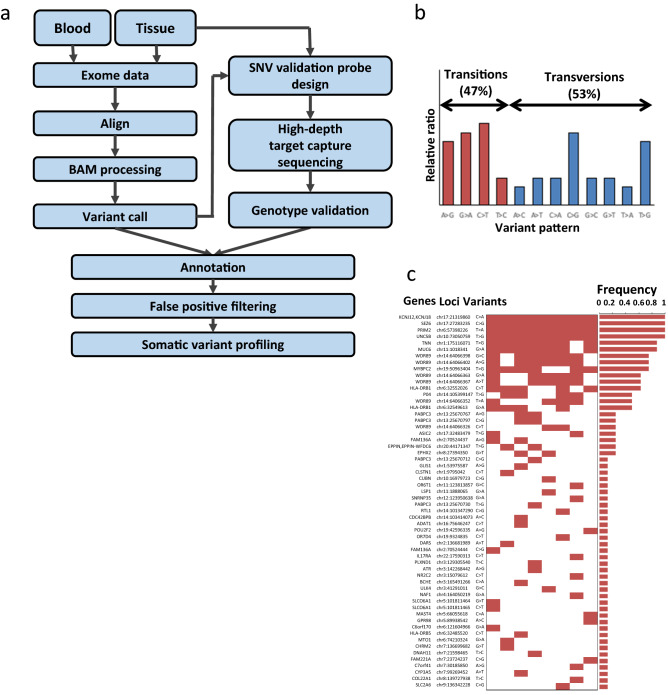
Somatic variant profiles of the discovery cohort. (**a**) Analysis pipeline used for whole-exome sequencing (WES) data (drawn in Microsoft 365 Powerpoint). (**b**) Relative ratio of transition and transversion variants (created in Microsoft 365 Excel; https://www.microsoft.com/ko-kr/microsoft-365). (**c**) Somatic variant profiles of the eight tissue-derived samples. Genes were sorted in descending order of frequency. The profile of the FFPE-derived sample was not plotted because of the low quality of the sequencing data. *NGDN* (p.S79C) confirmed by Sanger sequencing was considered the target of the validation cohort (created in Microsoft 365 Excel).

WES was performed to identify novel genes related to pure GH-secreting pituitary adenoma. Based on our aims, nine patients not carrying a variant at codon 201 or codon 227 of *GNAS* were selected for the cohort (Supplementary Table S1). Tumor tissue and blood samples were sequenced, and the data were analyzed with bioinformatics software (Fig. 2a).

Although the Ti/Tv ratio observed (47/53 = 0.89; Fig. 2b) was lower than the commonly reported ratio of ~ 2, Sanger sequencing of several mutant loci agreed with the data (Supplementary Fig. S1).

A total of 48 genes with 59 variants were confirmed for profiling (Fig. 2c). Additionally, whole genomic regions of seven genes (*HMGA2*, *FGFR4*, *PTTG1*, *RB1*, *GNAS*, *AIP*, *GPR101*) associated with acromegaly were added to the profiling target (Supplementary Table S2).

### Targeted resequencing

**Figure 3:**
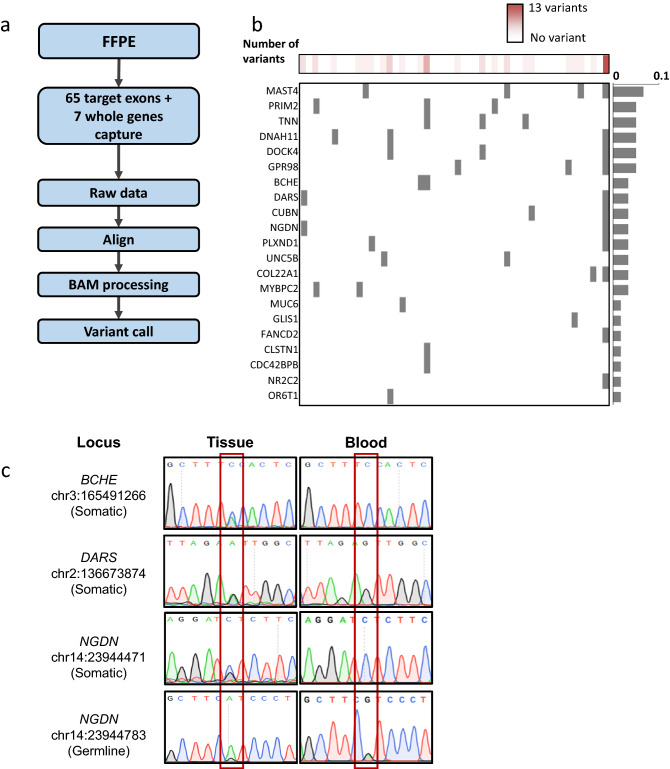
Variant profiles of the validation cohort. (**a**) Analysis pipeline used for targeted resequencing data (drawn in Microsoft 365 Powerpoint). (**b**) Variant profiles of validation cohort. Genes were sorted in descending order of frequency (created in Microsoft 365 Excel). (**c**) Representative images of Sanger sequencing of *DARS*, *BCHE*, and *NGDN*. Left and right panels represent the genotype of the tissue and blood, respectively (created in Snap Gene Viewer; https://www.snapgene.com/snapgene-viewer/).

We performed targeted sequencing to validate variants in the targeted genes (Fig. 3a). Among the 140 genomic DNA samples prepared from formalin-fixed, paraffin-embedded (FFPE) tissues, those carrying *GNAS* variant were confirmed by Sanger sequencing and excluded; 50 samples remained in the validation cohort. The samples were captured and sequenced to obtain high-depth sequencing data, which were analyzed to profile variants.

Twenty-six genes carrying variants were identified (Fig. 3b, Supplementary Fig. S2). *MAST4* was the most frequently mutated gene, found in 8% (4/50) of *GNAS*-negative patients. *PRIM2*, *TNN*, *STARD9*, *DNAH11*, *DOCK4*, and *GPR98* were found in 6% (3/50) of the cohort, and *BCHE*, *DARS*, *CUBN*, *NGDN*, *PLXND1*, *UNC5B*, and *COL22A1* were found in 4% (2/50) of the cohort. However, no variant was shared between any two samples, although frequently mutated genes were found.

### Statistical analysis of candidate variants

To investigate potential causal variants in the candidate genes found by WES and targeted resequencing, additional statistical analysis was performed. Variant-related Gene Ontology (GO) was analyzed using the Enrichr tool. The lists of genes found by WES and targeted resequencing were queried, and five high-ranked terms were compared (Supplementary Tables S3 and S4). Amyloid-beta binding (GO:0,001,540) terms in the molecular function category that are associated with GH-secreting pituitary adenomas^14^ were commonly found in WES and targeted resequencing, and *BCHE* and *CLSTN1* were related to the terms. Additionally, G-protein coupled receptor-related terms were observed in the WES results. G-protein coupled amine receptor activity (GO:0008227), G-protein coupled neurotransmitter receptor activity (GO:0099528), and G-protein coupled acetylcholine receptor activity (GO:0016907) terms were detected, with *CHRM2* and *OR6T1* related to the terms. Considering that *CHRM3* is a paralog of *CHRM2*^15^, we predicted that *CHRM2* may be important. However, only a small number of genes was related to the GO terms, whereas enriched GO terms analyzed from previously reported genes (Supplementary Table S5) resulted from a much larger number of related genes (Supplementary Table S6). Therefore, we analyzed the relationship between the variants and clinical manifestations to investigate whether the variants were associated with a phenotype, such as GH secretion.

The clinical manifestations of 50 patients selected for targeted resequencing are presented in Table 1. There was no significant difference in tumor size, age, sex, or Ki67 index according to the genetic variants. However, some variants were associated with the biochemical activities of GH-secreting pituitary adenoma, such as *BCHE*, *DARS*, *NGDN*, and *UNC5B* variants (Table 1). Although significance could not be calculated because of the limited number of subjects, the patients presented exceptionally increased GH and IGF-1 levels before transsphenoidal adenomectomy. Variants were validated by Sanger sequencing (Fig. 3c, Supplementary Fig. S3). The patients had no family history of pituitary adenomas or other neuroendocrine tumors.

**Table 1:** Clinical characteristics of patients with somatic variants in growth hormone (GH)-secreting pituitary adenoma.

	Total	NGDN		BCHE		DARS		UNC5B	
		S79C	R100H	P320L	W571C	V120G	T343I	V245M	S471C
Age (years old)	43 (24–72)	34	54	36	41	54	34	43	44
**Sex**									
Male	13 (26%)	0	1	0	1	1	0	0	1
Female	37 (74%)	1	0	1	0	0	1	1	0
**Hardy classification**									
I	12 (24%)	0	0	0	0	0	0	1	1
II	12 (24%)	0	0	0	0	0	0	0	0
III	12 (24%)	1	0	0	0	0	1	0	0
IV	14 (28%)	0	1	1	1	1	0	0	0
**Immunohistochemical staining**									
Focal GH	1 (2%)	0	0	0	0	0	0	0	0
GH	45 (90%)	1	1	1	1	1	1	1	1
Weak GH	4 (8%)	0	0	0	0	0	0	0	0
**Ki67 staining (%)**									
< 1	24 (48%)	0	0	0	0	0	0	1	1
1–2	10 (20%)	0	0	1	0	0	0	0	0
2–3	7 (14%)	0	1	0	0	1	0	0	0
Not checked	9 (18%)	1	0	0	1	0	1	0	0
**Preoperative OGTT**									
Basal GH (ng/dL)	15.0 (2.2–229.0)	139.2	109	19.29	21.8	109	139.2	3	59.13
Nadir GH (ng/mL)	11.0 (2.1–118.9)	118.9	41.4	17.29	15.7	41.4	118.9	2.1	44.1
IGF-1 (μg/L)	660.6 (355.3–1282.6)	971.5	907.8	884.4	814	907.8	971.5	355.3	1282.6

### Role of somatic variant in GH3 cells

**Figure 4:**
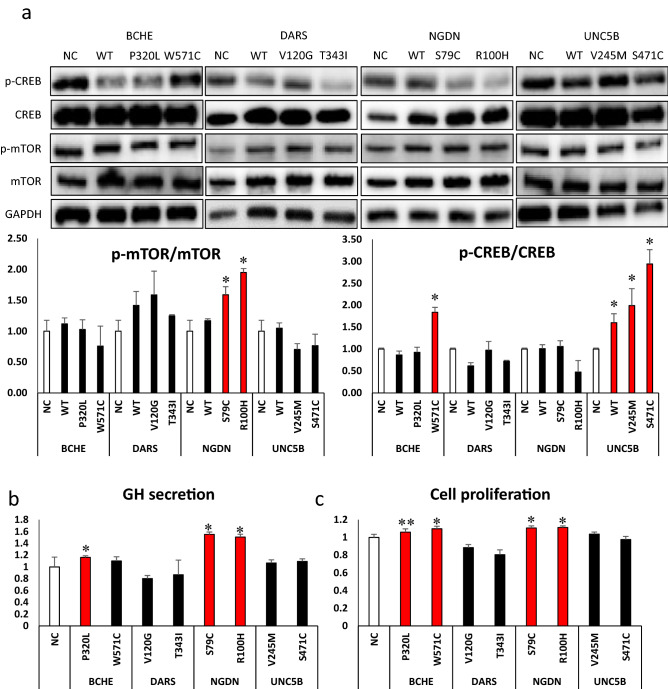
Biological role of somatic variants in GH3 cells. (**a**) Protein expression of genes responsible for growth hormone (GH) synthesis and secretion difference. Images of full-length blots are included in Supplementary Fig. S4 (created in Microsoft 365 Powerpoint, Excel). (**b**) GH secretion into the cell-culture media and (**c**) cell proliferation were also analyzed using transfected cells (created in Microsoft 365 Excel). All data are represented as the mean ± SE of triplicate experiments and representative of three independent experiments. Significant differences are denoted as *p < 0.05, **p < 0.01.

The results from WES and targeted resequencing and clinical results from GH-secreting pituitary adenoma patients confirmed that variants in *BCHE*, *DARS*, *NGDN*, and *UNC5B* may be involved in GH secretion. Therefore, in vitro experiments using rat GH-secreting GH3 cells were performed to investigate the effect of those gene variants on GH secretion. We detected the expression of proteins responsible for GH secretion (Fig. 4a) using GH3 cells transfected with each mutant of the selected genes (*BCHE*, *DARS*, *NGDN*, and *UNC5B*) and incubated the cells for 48 h. Phosphorylation of mTOR increased with transfection of *DARS* p.T343I (25%, *p* = 0.019), *NGDN* p.S79C (59%, *p* = 0.034), and *NGDN* p.R100H (95%, *p* = 0.031) mutants, and that of CREB increased with transfection of *BCHE* p.W571C (25%, *p* = 0.017), *UNC5B* p.V245M (99%, *p* = 0.031), and *UNC5B* p.S471C (194%, *p* = 0.002) mutants (Fig. 4a).

As transfection of mutants in GH3 cells significantly affected the signaling pathway of GH synthesis and secretion, we analyzed the concentration of GH secreted into the cell-culture media by enzyme-linked immunosorbent assay (ELISA). Among the several mutants, transfection of *BCHE* p.P320L (16%, *p*-value = 0.018), *NGDN* p.S79C (55%, *p* = 0.027), and *NGDN* p.R100H (50%, *p* = 0.047) mutants significantly increased GH secretion in cell-culture media compared with the vector used as a negative control (Fig. 4c). The amount of GH secreted in the media was normalized to the optical density values obtained for the same samples from the MTS assay (Fig. 4c). Furthermore, the effects of each mutant on cell proliferation were analyzed by MTS assay under the same experimental conditions. Compared with negative control-transfected cells, transfection of *BCHE* p.P320L (6%, *p* = 0.001), *BCHE* p.W571C (10%, *p* = 0.001), *NGDN* p.S79C (10%, *p* = 0.012), and *NGDN* p.R100H (11%, *p* = 0.030) mutants slightly increased cell proliferation (Fig. 4b). Based on the ELISA and MTS assays, the variants in *NGDN* and *BCHE* may affect GH secretion and tumor growth in GH-secreting pituitary adenomas. Finally, these variants were confirmed by Sanger sequencing with human GH-secreting pituitary adenoma (Fig. 3c, Supplementary Fig. S3).

## Discussion

In this study, we identified novel somatic variants associated with pure GH-secreting pituitary adenoma from patients with neither *GNAS* variants nor a family history. The following are the major findings of our study: (a) 48 genes with 59 somatic variants were identified among nine patients using WES; (b) targeted resequencing revealed variants in 26 recurrent genes, including in *MAST4*, *PRIM2*, *TNN*, *STARD9*, *DNAH11*, *DOCK4*, *GPR98*, *BCHE*, *DARS*, *CUBN*, *NGDN*, *PLXND1*, *UNC5B*, and *COL22A1*; (c) variants in previously reported genes, such as *HMGA2*, *FGFR4*, *PTTG1*, *RB1*, *GNAS*, *AIP*, and *GPR101*, were not detected by targeted resequencing; and (d) variants in *BCHE*, *DARS*, *NGDN*, and *UNC5B* were associated with increased biochemical activity of GH-secreting pituitary tumors.

In GO analysis, only a small number of genes was related to GO terms, whereas enriched GO terms for previously reported genes showed significant overlaps with pituitary adenomas and acromegaly-related terms (*p* = 1.3 × 10^–60^ and 1.6 × 10^–28^, respectively). This indicates that previously reported genes are rarely observed in other disease categories, and that current variants found in this study do not overlap with those found previously. Although previous databases included mostly western populations, only East Asians were analyzed in this study^11,15,16^. In several studies, ethnic differences were significantly associated with clinical manifestations and genetics of GH-secreting pituitary adenoma^17–19^. Furthermore, we analyzed pure GH-secreting pituitary adenomas without *GNAS* variant rather than general sporadic cases. The difference in a subject’s ethnicity and inclusion criteria may be responsible for the differences in the GO results. Also, while this manuscript was being reviewed, the variants were searched again in the recently updated COSMIC^20^ and ClinVar^21^databases and most of the variants have not been reported or rarely observed and not reported as significantly pathogenic (Supplementary Table S7).

The underlying pathophysiology of pituitary tumorigenesis is characterized by a combination of disordered cell proliferation concomitant with dysregulated hormone hypersecretion. GH-secreting pituitary adenoma formation results in unrestrained somatotroph proliferation associated with intrinsic cell cycle dysfunction as well as activation of cAMP signaling^1^. Most tumors are caused by heterogeneous variants, some of which are involved in the same molecular pathways and can promote tumor development^22^. Although variants with high frequency or hotspots, such as *GNAS*, were not identified, variants in recurrent genes among 50 GH-secreting pituitary adenomas suggested that these variants are involved in GH production from somatotroph cells. Among the gene lists of targeted resequencing, variants in 14 genes were observed in at least two or more patients. Regarding kinase activity, *MAST4*, *TNN*, *PLXND1*, and *COL22A1* are directly involved in the serine/threonine kinase family and PI3K-Akt and Akt signaling pathways, which can induce tumor formation in GH-secreting pituitary adenoma (www.genecards.org)^23–25^. Furthermore, several mutated genes can be modulated by transcription factors associated with GH-secreting pituitary adenomas. For example, *PRIM2*, *DOCK4*, and *DARS* can be modulated by STAT family proteins; *COL22A1*, *NGDN*, and *UNC5b* by GATA family proteins; and *DNAH11* by aryl hydrocarbon receptor and translocator, which induce somatotroph tumor formation (www.genecards.org).

GH secretion is controlled by Ca^2+^ and adenosine ATP signaling, and both signals appear to be frequently activated in GH-secreting pituitary adenomas. Activated cAMP and Ca^2+^ signaling lead to an increase in the cytosolic free calcium concentration in pituitary tumor cells^26,27^. Moreover, ATP, which is simultaneously released with pituitary hormones, induces an increase in the intracellular free calcium concentration in pituitary cells^28^. For example, Gsp variants induce constitutive activation of adenylate cyclase to convert ATP to cAMP. An increase in the intracellular cAMP concentration, in turn, leads to activation of protein kinase A and further mitogenic signaling in GH-secreting cells^8^. From a clinical perspective, *GNAS*-mutated GH-secreting pituitary adenomas present significantly increased biochemical activity among patients with acromegaly. Until now, genetic alterations associated with biochemical activity other than *GNAS* variants have not been reported. In this study, novel gene variants were identified in GH-secreting pituitary tumors and associated with biochemical activities. *BCHE* encodes butyryl cholinesterase and deacylate ghrelin, which stimulate GH secretion from somatotrophs^29^, and *DARS* encodes aspartyl-tRNA synthetase and is modulated by CXC chemokine receptor (CXCR) 7 in somatotrophs. Several studies reported that the expression of *CXCR7* is increased in prolactinoma, and that GH-secreting pituitary adenomas and *CXCR7* reduce the activity of *DARS*^30^. *NGDN* encodes the E1F4E binding protein and, in leukemia cell lines, *NGDN* knockdown activates the mTOR pathway^31^. *UNC5B* encodes a member of the netrin receptor family and is involved in angiogenesis. Furthermore, *UNC5B* was recently associated with aggressive behavior in null pituitary adenoma and silent subtype III pituitary corticotroph adenomas^32^. However, there have been no reports on these genetic variants in GH-secreting pituitary adenoma.

This study had several limitations. Although variants in recurrent genes were identified, functional studies of all variants were not performed. Nevertheless, several variants in four genes influencing the biochemical activities of GH-secreting pituitary adenoma were introduced in GH3 cells, and functional studies were performed. Second, the frequency of variants was relatively lower than that of previously reported somatic variants, such as *GNAS* variants in GH-secreting pituitary adenomas and *USP8* variants in Cushing’s disease. However, apart from *GNAS*, acromegaly-associated somatic variants are not well-established. Several studies have failed to demonstrate the clinical significance or recurrent variants of the reported somatic variants in sporadic GH-secreting pituitary tumors^11,15,16^. Furthermore, we found that variants actually promoted GH secretion in vitro in rat GH-secreting pituitary adenoma cells and human mutant vectors, showing high similarity between both human and rat sequences. Third, we excluded patients with *GNAS* variants and evaluated pure GH-expressing pituitary tumors in patients with acromegaly. In some conditions, GH-secreting pituitary adenomas are associated with somatic variants that combine with *GNAS,* except for hotspot variants; however, we sought to discover novel variants that function independently. Follow-up studies are needed to investigate the somatic variants found in this research. Finally, we did not evaluate granulation pattern of GH secreting pituitary tumors. Several studies reported that sparsely granulated type is associated with mutations in the extracellular domain of the growth hormone receptor and densely granulated type present more frequent *GNAS* mutation^33,34^. Further studies evaluating genetic alterations according to granulation type would be necessary.

In conclusion, several somatic variants were identified in sporadic pure GH-secreting pituitary adenomas from patients who had neither *GNAS* variants nor a family history. Furthermore, GH-secreting pituitary tumors with variants in *BCHE*, *DARS*, *NGDN*, and *UNC5B* presented high biochemical activities including GH and IGF-1. Although no novel recurrent point variants were identified, the observed somatic variants may affect pathways involved in tumorigenesis and biochemical activities of GH-secreting pituitary adenomas. Further studies are needed to determine the functional mechanism of variants in selected genes that affect GH secretion and pituitary adenoma formation.

## Methods

### Patients

Among patients in the Severance Hospital Pituitary Tumor Cohort, 232 subjects received transsphenoidal adenomectomy following a diagnosis of GH-secreting pituitary adenoma from January 2012 to January 2017 at Severance Hospital, Yonsei University College of Medicine, Seoul, Korea. All the enrolled patients were single ethnicity, Korean. GH-secreting pituitary tumors were confirmed by measuring serum insulin-like growth factor-1 (IGF-1) and nadir GH levels during the 75-g oral glucose tolerance test. Immunohistochemical staining of the operated pituitary tumors was performed for GH, prolactin, luteinizing hormone, follicle-stimulating hormone, thyroid stimulating hormone, and adrenocorticotropic hormone. Pituitary tumors expressing hormones other than GH were excluded from this study. Finally, the tumor tissues of 140 patients with acromegaly expressing only GH were evaluated. No patients had a family history of pituitary adenoma. Pituitary tumors were classified based on dynamic magnetic resonance imaging of the sella turcica and parasellar region according to the modified Hardy radiological classifications, as described previously^35^.

Immunoradiometric assay (hGH-RIACT; Cis Bio International, Gif-sur-Yvette, France) was used to measure the GH concentration. This assay had an analytical sensitivity of 0.03 μIU/mL, within-assay coefficient of variation (CV) of 1.3–2.1%, and inter-assay CV of 3.8–5.0%. The World Health Organization international standard (WHO IS 98/574) was used to classify GH values. An immunoradiometric assay system (IGF-1 NEXT IRMA CT; Biocode-Hycel, Liège, Belgium) was used to measure the IGF-1 concentration. The minimum detectable IGF-1 concentration was 1.25 ng/L, within-assay CV was 2.6–4.4%, and inter-assay CV was 7.4–9.1%^36^.

This study was conducted in accordance with the Declaration of Helsinki and approved by our institutional review board and Ethics Committee (Yonsei University Institutional Review Board and Ethics Committee: approval # 4-2011-0740). Written informed consent was required and obtained from all patients.

### WES

For WES, 29 patients agreed to tissue collection during transsphenoidal adenomectomy. Genomic DNA samples from fresh frozen tissue were extracted using a DNeasy Blood & Tissue Kit (Qiagen, Hilden, Germany). Variants at codons 201 and 227 in *GNAS* were tested by PCR followed by Sanger sequencing (Macrogen, Seoul, Korea). PCR and Sanger sequencing primers were designed to cover both hotspot variants of *GNAS* (Supplementary Table S8). Eight *GNAS* variant-negative pituitary tumor samples and one additional sample prepared from FFPE tissue were subjected to WES, and each normal genomic DNA sample from blood was extracted using a DNeasy Blood & Tissue Kit (Qiagen). Shotgun DNA libraries for sequencing were constructed, and WES was performed (Celemics, Seoul, Korea) using the SureSelect Human All Exon V4 + UTRs kit (Agilent Technologies, Santa Clara, CA, USA). Each library was pooled and sequenced with a Hiseq2500 (Illumina, San Diego, CA, USA).

### Analysis of exome data

Raw sequencing data (FASTQ) were aligned to the human genome (NCBI Build 37; hg19) to generate a SAM format file using Novoalign (v. 2.07.18, Novocraft; http://www.novocraft.com/products/novoalign/). SAM files were converted to BAM format using SAMtools (v. 0.1.18, SAMtools; http://www.htslib.org/)^37^, and each expanded data of tissue sample was merged into one BAM file. To modify the BAM files, duplications were removed using Picard v1.67 MarkDuplicates (Picard Tools; http://broadinstitute.github.io/picard/), and indels were fixed around a known indel position using built-in modules for indel realignment in Genome Analysis Tool Kit (GATK, v. 2.7.2, Broad Institute; https://gatk.broadinstitute.org/hc/en-us)^38^. Base quality was recalibrated using modules for base quality recalibration in GATK.

Variants were called from modified BAM files using muTect (v. 1.1.4, Broad Institute; https://software.broadinstitute.org/cancer/cga/mutect)^39^ and Varscan2 (v. 2.2.11, http://dkoboldt.github.io/varscan/)^40^, annotated using Table_ANNOVAR with screening variants from several databases; Exome Sequencing Project 6500 (ESP6500), 1000 Genome Project (1000GP), Cosmic, ClinVar, and functional prediction were filtered using PolyPhen-2 and avsift algorithms. We filtered variants with an allelic frequency > 1% in the database.

### Targeted resequencing and data analysis

For single nucleotide variant (SNV) validation, probes were designed to cover 60 bp on either side of the targeted SNV, synthesized from microarray, and in vitro-transcribed to contain biotinylated UTP using a MAXIscript™ T7 Transcription Kit (Thermo Fisher Scientific, Waltham, MA, USA). Target regions were captured from tumor tissue samples used in exome sequencing, which was performed at Celemics, Inc.

Raw sequencing data (FASTQ) were aligned using Novoalign (v2.07.18). SAM files were converted to BAM format using SAMtools (v. 0.1.18). Each genotype of target loci was piled-up using the *mpileup* command in SAMtools and compared to exome sequencing data. Variants with a variant allele frequency > 0.1, total depth > 15, and variant depth > 2 were selected.

For target profiling, 120-bp probes were designed to cover the targeted region with 2 × tiling density (almost all probes half-overlapped with the nearest probe). The probes were prepared as described for the SNV validation panel. Genomic DNA samples were extracted from FFPE samples using the QIAamp DNA FFPE Tissue Kit (Qiagen) and used to construct the NGS library with a SPARK™ DNA Sample Prep Kit (Enzymatics, Beverly, MA, USA); capture was performed at Celemics, Inc. Sequencing data were aligned and modified as described for exome sequencing analysis, and variants were called using the UnifiedGenotyper command in GATK.

### Gene set enrichment analysis

GO was analyzed using the Enrichr tool (https://amp.pharm.mssm.edu/Enrichr/)^41^. A list of mutant genes was queried for four categories: “Rare Diseases GeneRIF Gene Lists,” “GO Biological Process2018,” “GO Cellular Component 2018,” and “GO Molecular Function 2018.” Enriched terms with a p-value < 0.05 were retrieved and ranked in ascending order.

### Cell culture

GH3 cells (American Type Culture Collection, Manassas, VA, USA) were cultured in high-glucose Dulbecco’s modified Eagle’s medium (Hyclone, Logan, UT, USA) supplemented with 4 mM l-glutamine, 10% fetal bovine serum (Hyclone), and penicillin–streptomycin solution (Hyclone) in a humidified atmosphere with 5% CO_2_ and 95% air at 37 °C.

### Transfection of mutant plasmids

pcDNA3.1-FLAG- *BCHE*, *DARS*, *NGDN*, or *UNC5B* wild-type (WT) was purchased from Origene (Rockville, MD, USA), and each mutant plasmid of target genes was generated with Site-Directed Mutagenesis Kits according to the manufacturer’s protocol (Thermo Fisher Scientific). To determine the effects of each variant on cancer cell properties, GH3 cells were transfected with pcDNA3.1 control vector, pcDNA3.1-FLAG-WT of target genes, or pcDNA3.1-FLAG-mutant of target genes using Polyjet reagent (Invitrogen, Carlsbad, CA, USA). The cells were analyzed by quantitative real-time polymerase chain reaction (qRT-PCR), western blotting, MTS assay (Promega, #G3582, Madison, WI, USA), and ELISA for GH (Merck, Kenilworth, NJ, USA) at 48 h after transfection^42^.

### qRT-PCR

qRT-PCR was performed using a SYBR Green Reverse Transcription Kit (Applied Biosystems, Foster City, CA, USA) according to the manufacturer’s protocols, using the following primers: rat Gh forward, 5′-CAAAGAGTTCGAGCGTGCCTA-3′, and reverse, 5′-TGGGATGGTCTCTGAGAAGCA-3′, rat Gapdh forward, 5′- GGATGGAATTGTGAGGGAGA-3′, and reverse, 5′-GAGGACCAGGTTGTCTCCTG-3′.

### Western blot analysis

GH3 cells in 10-cm plates were lysed in radio-immunoprecipitation assay buffer (20 mM Tris, 2 mM EDTA, 150 mM NaCl, 0.5% Triton X-100, 5% phosphatase inhibitors, 0.5% protease inhibitors), and the protein content was measured using a Coomassie (Bradford, UK) Protein Assay Kit (Pierce, Rockford, IL, USA). Equal amounts of protein (10 mg) were heat-denatured in 2 × sample buffer (2% SDS, 62.5 mM Tris, pH 6.8, 0.01% bromophenol blue, 1.43 mM mercaptoethanol, and 0.1% glycerol), separated on a 10% SDS–polyacrylamide gel, transferred onto polyvinylidene difluoride membranes (Bio-Rad), and blotted with the appropriate antibodies: anti-GH (Santa Cruz Biotechnology, Dallas, TX, USA), anti-tAKT (Cell Signaling Technology, Danvers, MA, USA), anti-pAKT (Cell Signaling Technology), anti-mTOR (Cell Signaling Technology), anti-pCREB (Cell Signaling Technology), anti-tCREB (Cell Signaling Technology), and anti-β-actin (Santa Cruz Biotechnology). Immunocomplexes were detected using an enhanced chemiluminescence system (Cell Signaling Technology)^43^.

### Statistical analysis

Continuous and categorical variables are expressed as the medians (range). The data were analyzed using the SPSS software package for Windows (v. 25.0, IBM; https://www.ibm.com/kr-ko/analytics/spss-statistics-software). The Mann–Whitney U test and Kruskal–Wallis test were used for continuous variables, and linear-by-linear association for categorical variables was performed as appropriate. All statistical tests were two-tailed, and a *p*-value < 0.05 was considered significant^36^.

## Supplementary Information

Supplementary Information.

## Data Availability

All data generated or analyzed during this study are included in this published article or in the data repositories listed in References.

## References

[1] Melmed S (2009). Acromegaly pathogenesis and treatment. J Clin Invest.

[2] Dekkers OM, Biermasz NR, Pereira AM, Romijn JA, Vandenbroucke JP (2008). Mortality in acromegaly: A metaanalysis. J. Clin. Endocrinol. Metab..

[3] Maison P, Tropeano A, Macquin Mavier I, Giustina A, Chanson P (2007). Impact of somatostatin analogs on the heart in acromegaly: A metaanalysis. J. Clin. Endocrinol. Metab..

[4] Asa SL, Ezzat S (2002). The pathogenesis of pituitary tumours. Nat. Rev. Cancer.

[5] Clayton RN, Farrell WE (2004). Pituitary tumour clonality revisited. Front. Horm. Res..

[6] Landis CA, Harsh G, Lyons J, Davis RL, McCormick F, Bourne HR (1990). Clinical characteristics of acromegalic patients whose pituitary tumors contain mutant Gs protein. J. Clin. Endocrinol. Metab..

[7] Landis CA, Masters SB, Spada A, Pace AM, Bourne HR, Vallar L (1989). GTPase inhibiting mutations activate the alpha chain of Gs and stimulate adenylyl cyclase in human pituitary tumours. Nature.

[8] Vallar L, Spada A, Giannattasio G (1987). Altered Gs and adenylate cyclase activity in human GH-secreting pituitary adenomas. Nature.

[9] Makinen N, Mehine M, Tolvanen J, Kaasinen E, Li Y, Lehtonen HJ, Gentile M, Yan J, Enge M, Taipale M, Aavikko M, Katainen R, Virolainen E, Bohling T, Koski TA, Launonen V, Sjoberg J, Taipale J, Vahteristo P, Aaltonen LA (2011). MED12, the mediator complex subunit 12 gene, is mutated at high frequency in uterine leiomyomas. Science.

[10] Cancer Genome Atlas Network (2012). Comprehensive molecular characterization of human colon and rectal cancer. Nature.

[11] Valimaki N, Demir H, Pitkanen E, Kaasinen E, Karppinen A, Kivipelto L, Schalin-Jantti C, Aaltonen LA, Karhu A (2015). Whole-Genome sequencing of growth hormone (GH)-secreting pituitary adenomas. J. Clin. Endocrinol. Metab..

[12] Newey PJ, Nesbit MA, Rimmer AJ, Head RA, Gorvin CM, Attar M, Gregory L, Wass JA, Buck D, Karavitaki N, Grossman AB, McVean G, Ansorge O, Thakker RV (2013). Whole-exome sequencing studies of nonfunctioning pituitary adenomas. J. Clin. Endocrinol. Metab..

[13] Reincke M, Sbiera S, Hayakawa A, Theodoropoulou M, Osswald A, Beuschlein F, Meitinger T, Mizuno-Yamasaki E, Kawaguchi K, Saeki Y, Tanaka K, Wieland T, Graf E, Saeger W, Ronchi CL, Allolio B, Buchfelder M, Strom TM, Fassnacht M, Komada M (2015). Mutations in the deubiquitinase gene USP8 cause Cushing's disease. Nat. Genet..

[14] Mori H, Mori S, Saitoh Y, Moriwaki K, Iida S, Matsumoto K (1985). Growth hormone-producing pituitary adenoma with crystal-like amyloid immunohistochemically positive for growth hormone. Cancer.

[15] Ronchi CL, Peverelli E, Herterich S, Weigand I, Mantovani G, Schwarzmayr T, Sbiera S, Allolio B, Honegger J, Appenzeller S, Lania AG, Reincke M, Calebiro D, Spada A, Buchfelder M, Flitsch J, Strom TM, Fassnacht M (2016). Landscape of somatic mutations in sporadic GH-secreting pituitary adenomas. Eur. J. Endocrinol..

[16] Trivellin G, Daly AF, Faucz FR, Yuan B, Rostomyan L, Larco DO, Schernthaner-Reiter MH, Szarek E, Leal LF, Caberg JH, Castermans E, Villa C, Dimopoulos A, Chittiboina P, Xekouki P, Shah N, Metzger D, Lysy PA, Ferrante E, Strebkova N, Mazerkina N, Zatelli MC, Lodish M, Horvath A, de Alexandre RB, Manning AD, Levy I, Keil MF, Sierra Mde L, Palmeira L, Coppieters W, Georges M, Naves LA, Jamar M, Bours V, Wu TJ, Choong CS, Bertherat J, Chanson P, Kamenicky P, Farrell WE, Barlier A, Quezado M, Bjelobaba I, Stojilkovic SS, Wess J, Costanzi S, Liu P, Lupski JR, Beckers A, Stratakis CA (2014). Gigantism and acromegaly due to Xq26 microduplications and GPR101 mutation. N. Engl. J. Med..

[17] Yoshimoto K, Iwahana H, Fukuda A, Sano T, Itakura M (1993). Rare mutations of the Gs alpha subunit gene in human endocrine tumors. Mutation detection by polymerase chain reaction-primer-introduced restriction analysis. Cancer.

[18] Cai F, Zhang YD, Zhao X, Yang YK, Ma SH, Dai CX, Liu XH, Yao Y, Feng M, Wei JJ, Xing B, Jiao YH, Wei ZQ, Yin ZM, Zhang B, Gu F, Wang RZ (2013). Screening for AIP gene mutations in a Han Chinese pituitary adenoma cohort followed by LOH analysis. Eur. J. Endocrinol..

[19] Gao M, Zhu B, Xu Z, Liu S, Liu J, Zhang G, Gao Y, Fan Y, Kang X (2018). Association between acromegaly and a single nucleotide polymorphism (rs2854744) in the IGFBP3 gene. BMC Med. Genet..

[20] Tate JG, Bamford S, Jubb HC, Sondka Z, Beare DM, Bindal N, Boutselakis H, Cole CG, Creatore C, Dawson E, Fish P, Harsha B, Hathaway C, Jupe SC, Kok CY, Noble K, Ponting L, Ramshaw CC, Rye CE, Speedy HE, Stefancsik R, Thompson SL, Wang S, Ward S, Campbell PJ, Forbes SA (2018). COSMIC: The catalogue of somatic mutations in cancer. Nucleic Acids Res..

[21] Landrum MJ, Chitipiralla S, Brown GR, Chen C, Gu B, Hart J, Hoffman D, Jang W, Kaur K, Liu C, Lyoshin V, Maddipatla Z, Maiti R, Mitchell J, O'Leary N, Riley GR, Shi W, Zhou G, Schneider V, Maglott D, Holmes JB, Kattman BL (2020). ClinVar: Improvements to accessing data. Nucleic Acids Res..

[22] Wood LD, Parsons DW, Jones S, Lin J, Sjoblom T, Leary RJ, Shen D, Boca SM, Barber T, Ptak J, Silliman N, Szabo S, Dezso Z, Ustyanksky V, Nikolskaya T, Nikolsky Y, Karchin R, Wilson PA, Kaminker JS, Zhang Z, Croshaw R, Willis J, Dawson D, Shipitsin M, Willson JK, Sukumar S, Polyak K, Park BH, Pethiyagoda CL, Pant PV, Ballinger DG, Sparks AB, Hartigan J, Smith DR, Suh E, Papadopoulos N, Buckhaults P, Markowitz SD, Parmigiani G, Kinzler KW, Velculescu VE, Vogelstein B (2007). The genomic landscapes of human breast and colorectal cancers. Science.

[23] Charvet B, Guiraud A, Malbouyres M, Zwolanek D, Guillon E, Bretaud S, Monnot C, Schulze J, Bader HL, Allard B, Koch M, Ruggiero F (2013). Knockdown of col22a1 gene in zebrafish induces a muscular dystrophy by disruption of the myotendinous junction. Development.

[24] Sun L, Gu S, Li X, Sun Y, Zheng D, Yu K, Ji C, Tang R, Xie Y, Mao Y (2006). Identification of a novel human MAST4 gene, a new member of the microtubule associated serine-threonine kinase family. Mol. Biol. (Mosk).

[25] Cariboni A, Andre V, Chauvet S, Cassatella D, Davidson K, Caramello A, Fantin A, Bouloux P, Mann F, Ruhrberg C (2015). Dysfunctional SEMA3E signaling underlies gonadotropin-releasing hormone neuron deficiency in Kallmann syndrome. J. Clin. Invest..

[26] Meier K, Knepel W, Schofl C (1988). Potassium depolarization elevates cytosolic free calcium concentration in rat anterior pituitary cells through 1,4-dihydropyridine-sensitive, omega-conotoxin-insensitive calcium channels. Endocrinology.

[27] Garcia A, Alvarez CV, Smith RG, Dieguez C (2001). Regulation of Pit-1 expression by ghrelin and GHRP-6 through the GH secretagogue receptor. Mol. Endocrinol..

[28] Chen ZP, Kratzmeier M, Levy A, McArdle CA, Poch A, Day A, Mukhopadhyay AK, Lightman SL (1995). Evidence for a role of pituitary ATP receptors in the regulation of pituitary function. Proc. Natl. Acad. Sci. USA.

[29] Chen VP, Gao Y, Geng L, Brimijoin S (2017). Butyrylcholinesterase regulates central ghrelin signaling and has an impact on food intake and glucose homeostasis. Int. J. Obes. (Lond.).

[30] Yoshida D, Nomura R, Teramoto A (2009). Signalling pathway mediated by CXCR7, an alternative chemokine receptor for stromal-cell derived factor-1alpha, in AtT20 mouse adrenocorticotrophic hormone-secreting pituitary adenoma cells. J. Neuroendocrinol..

[31] Chen K, Lu S, Cheng H, Tang G, Liu M, Zhou H, Wang J (2015). High expression of neuroguidin increases the sensitivity of acute myeloid leukemia cells to chemotherapeutic drugs. J. Hematol. Oncol..

[32] Richardson TE, Shen Z-J, Kanchwala M, Xing C, Filatenkov A, Shang P, Barnett S, Abedin Z, Malter JS, Raisanen JM, Burns DK, White CL, Hatanpaa KJ (2017). Aggressive behavior in silent subtype III pituitary adenomas may depend on suppression of local immune response: A whole transcriptome analysis. J. Neuropathol. Exp. Neurol..

[33] Larkin S, Reddy R, Karavitaki N, Cudlip S, Wass J, Ansorge O (2013). Granulation pattern, but not GSP or GHR mutation, is associated with clinical characteristics in somatostatin-naive patients with somatotroph adenomas. Eur. J. Endocrinol..

[34] Gadelha MR, Kasuki L, Korbonits M (2013). Novel pathway for somatostatin analogs in patients with acromegaly. Trends Endocrinol. Metab..

[35] Ku CR, Kim EH, Oh MC, Lee EJ, Kim SH (2012). Surgical and endocrinological outcomes in the treatment of growth hormone-secreting pituitary adenomas according to the shift of surgical paradigm. Neurosurgery.

[36] Ku CR, Hong JW, Kim EH, Kim SH, Lee EJ (2014). Clinical predictors of GH deficiency in surgically cured acromegalic patients. Eur. J. Endocrinol..

[37] Li H, Handsaker B, Wysoker A, Fennell T, Ruan J, Homer N, Marth G, Abecasis G, Durbin R, Subgroup GPDP (2009). The Sequence Alignment/Map format and SAMtools. Bioinformatics.

[38] McKenna A, Hanna M, Banks E, Sivachenko A, Cibulskis K, Kernytsky A, Garimella K, Altshuler D, Gabriel S, Daly M, DePristo MA (2010). The Genome Analysis Toolkit: A MapReduce framework for analyzing next-generation DNA sequencing data. Genome Res..

[39] Cibulskis K, Lawrence MS, Carter SL, Sivachenko A, Jaffe D, Sougnez C, Gabriel S, Meyerson M, Lander ES, Getz G (2013). Sensitive detection of somatic point mutations in impure and heterogeneous cancer samples. Nat. Biotechnol..

[40] Koboldt DC, Zhang Q, Larson DE, Shen D, McLellan MD, Lin L, Miller CA, Mardis ER, Ding L, Wilson RK (2012). VarScan 2: Somatic mutation and copy number alteration discovery in cancer by exome sequencing. Genome Res..

[41] Chen EY, Tan CM, Kou Y, Duan Q, Wang Z, Meirelles GV, Clark NR, Ma'ayan A (2013). Enrichr: Interactive and collaborative HTML5 gene list enrichment analysis tool. BMC Bioinform..

[42] Lee WK, Lee SG, Yim SH, Kim D, Kim H, Jeong S, Jung SG, Jo YS, Lee J (2018). Whole exome sequencing identifies a novel hedgehog-interacting protein G516R mutation in locally advanced papillary thyroid cancer. Int. J. Mol. Sci..

[43] Lee Y, Kim JM, Lee EJ (2008). Functional expression of CXCR4 in somatotrophs: CXCL12 activates GH gene, GH production and secretion, and cellular proliferation. J. Endocrinol..

