# Supplementary material for: Novel somatic variants involved in biochemical activity of pure growth hormone-secreting pituitary adenoma without GNAS variant
Source: Sci Rep. 2021 Aug 16;11:16530. doi: 10.1038/s41598-021-95829-3 (PMC8368009; doi:10.1038/s41598-021-95829-3)
Supplement: Supplementary file 1 — Supplementary Information. [file 41598_2021_95829_MOESM1_ESM.docx]

| **Supplementary Table S1. Clinical characteristics of the cohort evaluated by whole-exome sequencing** | | | | | | | |  |
| --- | --- | --- | --- | --- | --- | --- | --- | --- |
| **No.** | **Age** | **Sex** | **Hardy** | **IHC** | **Ki-67** | **Basal GH (ng/mL)** | **Nadir GH in OGTT (ng/mL)** | **IGF-1 (ng/mL)** |
| 1 | 56 | F | 2 | GH | <1% | 4.6 | 4.6 | 570.6 |
| 2 | 58 | F | 2 | GH | <1% | 47.2 | 47.2 | 658.7 |
| 3 | 38 | F | 3 | GH | 1% | 58.2 | 33.80 | 827.4 |
| 4 | 42 | F | 3 | GH | 1-2% | 17 | 15.50 | 558.1 |
| 5 | 52 | F | 4 | weak GH | <1% | 5.3 | 2.80 | 456.9 |
| 6 | 35 | F | 3 | GH | 1-2% | 121 | 105 | 725.5 |
| 7 | 37 | M | 1 | GH | <1% | 7.5 | 6.30 | 675.0 |
| 8 | 39 | M | 2 | GH | <1% | 5.7 | 4.9 | 1490 |
| 9 | 39 | M | 4 | GH | 2-3% | 70.8 | 70.80 | 774.1 |

| **Supplementary Table S2. Genes used for targeted resequencing** | |
| --- | --- |
| **Target region** | **Genes for targeted resequencing** |
| Exons | *KCNJ12 (KCNJ18), SEZ6, PRIM2, UNC5B, TNN, MUC6, WDR89, MYBPC2, HLA-DRB1, P04, PABPC3, ASIC2, FAM136A, EPPIN,* *EPPIN-WFDC6, EPHX2, GLIS1, CLSTN1, CUBN, OR6T1, LSP1, SNRNP35, RTL1, CDC42BPB, ADAT1, POU2F2, OR7D4, DARS, IL17RA, PLXND1, ATR, NR2C2, BCHE, ULK4, NAF1, SLCO6A1, MAST4, GPR98, C6orf170, HLA-DRB5, MTO1, CHRM2, DNAH11, FAM221A, C7orf41, CYP3A5, COL22A1, SLC2A6, NGDN* |
| Whole gene | *HMGA2, FGFR4, PTTG1, RB1, GNAS, AIP, GPR101* |

| **Supplementary Table S3. Enrichment results for genes found by whole-exome sequencing** | | | | |
| --- | --- | --- | --- | --- |
| **Gene_set** | **Rank** | **Description** | **p_value** | **Genes** |
| Rare_Diseases_GeneRIF_Gene_Lists | 1 | Alveolar_echinococcosis | 5.57E-04 | [HLA-DRB5, HLA-DRB1] |
|  | 2 | Papillon_Lefevre_syndrome | 5.57E-04 | [NAF1, HLA-DRB1] |
|  | 3 | Geographic_tongue | 5.57E-04 | [CYP3A5, HLA-DRB1] |
|  | 4 | Diffuse_panbronchiolitis | 1.00E-03 | [HLA-DRB5, HLA-DRB1] |
|  | 5 | Lewy_body_dementia | 1.02E-03 | [CHRM2, BCHE, HLA-DRB5, HLA-DRB1] |
| GO_Biological_Process_2018 | 1 | regulation of neuron migration (GO:2001222) | 9.01E-04 | [TNN, ULK4] |
|  | 2 | lipid modification (GO:0030258) | 1.83E-03 | [EPHX2, CYP3A5] |
|  | 3 | positive regulation of telomere maintenance via telomerase (GO:0032212) | 2.72E-03 | [NAF1, ATR] |
|  | 4 | positive regulation of telomere maintenance via telomere lengthening (GO:1904358) | 3.06E-03 | [NAF1, ATR] |
|  | 5 | regulation of blood circulation (GO:1903522) | 3.42E-03 | [CHRM2, KCNJ12] |
| GO_Cellular_Component_2018 | 1 | MHC class II protein complex (GO:0042613) | 4.83E-04 | [HLA-DRB5, HLA-DRB1] |
|  | 2 | MHC protein complex (GO:0042611) | 8.07E-04 | [HLA-DRB5, HLA-DRB1] |
|  | 3 | clathrin-coated vesicle membrane (GO:0030665) | 9.46E-04 | [CHRM2, HLA-DRB5, HLA-DRB1] |
|  | 4 | integral component of lumenal side of endoplasmic reticulum membrane (GO:0071556) | 2.25E-03 | [HLA-DRB5, HLA-DRB1] |
|  | 5 | clathrin-coated endocytic vesicle membrane (GO:0030669) | 2.89E-03 | [HLA-DRB5, HLA-DRB1] |
| GO_Molecular_Function_2018 | 1 | amyloid-beta binding (GO:0001540) | 6.16E-03 | [BCHE, CLSTN1] |
|  | 2 | G-protein coupled amine receptor activity (GO:0008227) | 7.95E-03 | [CHRM2, OR6T1] |
|  | 3 | ligand-gated cation channel activity (GO:0099094) | 9.06E-03 | [KCNJ12, ASIC2] |
|  | 4 | G-protein coupled neurotransmitter receptor activity (GO:0099528) | 1.40E-02 | [CHRM2] |
|  | 5 | G-protein coupled acetylcholine receptor activity (GO:0016907) | 1.40E-02 | [CHRM2] |

| **Supplementary Table S4. Enrichment results for genes found in targeted resequencing** | | | | |
| --- | --- | --- | --- | --- |
| **Gene_set** | **Rank** | **Description** | **p_value** | **Genes** |
| Rare_Diseases_GeneRIF_Gene_Lists | 1 | Secretory_breast_carcinoma | 8.37E-03 | [MUC6] |
|  | 2 | Charlie_M_syndrome | 1.67E-02 | [PLXND1] |
|  | 3 | Germinoma | 1.83E-02 | [FANCD2, NR2C2] |
|  | 4 | Moebius_syndrome | 1.98E-02 | [PLXND1] |
|  | 5 | Anemia_due_to_Adenosine_triphosphatase_deficiency | 1.98E-02 | [FANCD2] |
| GO_Biological_Process_2018 | 1 | actomyosin structure organization (GO:0031032) | 2.57E-03 | [MYBPC2, CDC42BPB] |
|  | 2 | negative regulation of osteoblast proliferation (GO:0033689) | 7.33E-03 | [TNN] |
|  | 3 | cytoskeleton organization (GO:0007010) | 7.76E-03 | [MAST4, CDC42BPB] |
|  | 4 | regulation of vascular associated smooth muscle cell migration (GO:1904752) | 8.37E-03 | [DOCK4] |
|  | 5 | osteoblast development (GO:0002076) | 8.37E-03 | [TNN] |
| GO_Cellular_Component_2018 | 1 | alpha DNA polymerase:primase complex (GO:0005658) | 6.28E-03 | [PRIM2] |
|  | 2 | extrinsic component of external side of plasma membrane (GO:0031232) | 7.33E-03 | [CUBN] |
|  | 3 | nuclear replisome (GO:0043601) | 1.77E-02 | [PRIM2] |
|  | 4 | striated muscle thin filament (GO:0005865) | 1.98E-02 | [MYBPC2] |
|  | 5 | brush border membrane (GO:0031526) | 3.00E-02 | [CUBN] |
| GO_Molecular_Function_2018 | 1 | amyloid-beta binding (GO:0001540) | 1.25E-03 | [BCHE, CLSTN1] |
|  | 2 | semaphorin receptor activity (GO:0017154) | 1.15E-02 | [PLXND1] |
|  | 3 | DNA polymerase binding (GO:0070182) | 1.67E-02 | [FANCD2] |
|  | 4 | muscle alpha-actinin binding (GO:0051371) | 1.67E-02 | [MYBPC2] |
|  | 5 | ATP-dependent microtubule motor activity, minus-end-directed (GO:0008569) | 1.77E-02 | [DNAH11] |

| **Supplementary Table S5. Genes reported in previous studies** | |
| --- | --- |
| **Journal title** | **Genes** |
| Biomarkers of aggressive pituitary adenomas | FGFR4, FGFR2, MAGEA3, MMP1, MMP9, MMP2, MMP14, GHR, CRMP1, ADAMTS6, PTTG1, CCNB1, AURKB, ASK, CENPE, 11p, CD44, DGKZ, TSG101, GTF2H1, HTATIP2, MEN1, HMGA2 |
| Pathogenesis of pituitary tumors | GNAS, CREB, AIP, MEN1, PRKAR1A, HRAS, CCNB2, CCND1, CDKN1B, HMGA2, FGFR4, PTTG1, RB1, CDKN2A, SMARCA4, GADD45G, MEG3 |
| Pituitary adenoma pathogenesis: an update | MEN1, PRKAR1A, AIP, CDKN1B, GNAS, DKC1, HMGA1, HMGA2, PITX2, MAGEA3, MEG3, GADD45G, RASSF1 |
| The genetics of pituitary adenomas | RB1, CCND1, CCND3, CCNE1, CCNA1, CDKN2B, CDKN2A, CDKN2C, CDKN1A, PTTG1, PLAGL1, GADD45G, FGFR4, FGFR2, MAGEA3, BMP4, EGFR, PRKCA, PIK3CA, KRAS, HRAS, NRAS, WIF1, MEG3, RHBDD3, FOLR1, FOLR2, FOLR3, MERTK, ODC1, LAPTM4B, BAG1, GNAS, MEN1, CDKN1B, PRKAR1A, AIP |
| The pituitary tumour epigenome: aberrations and prospects for targeted therapy | GNAS, MAGEA3, DNMT3B, CDKN2A, DNMT3B, CDKN2A, RB1, DAPK1, MEG3, GADD45G, RHBDD3, NNAT, FGFR2 |
| Gigantism and Acromegaly Due to Xq26 Microduplications and GPR101 Mutation | GPR101 |
| Mutations in the deubiquitinase gene USP8 cause Cushing’s disease | USP8 |
| Germline and Somatic DICER1 Mutations in a Pituitary Blastoma Causing Infantile-Onset Cushing’s Disease | DICER1 |
| Genetic Predictors of Response to Different Medical Therapies in Acromegaly | GNAS, AIP, ZAC1, GHR, IGF1, IGFBP3, UGT1A1 |
| The Epigenomic Landscapeof PituitaryAdenomas Reveals Specific Alterations and Differentiates Among Acromegaly, Cushing's Disease and Endocrine-Inactive Subtypes | GNAS, USP8, PABPC1 |
| Genome-wide scan identifies novel modifier loci of acromegalic phenotypes for isolated familial somatotropinoma | AIP |
| Epidemiology and etiopathogenesis of pituitary adenomas | CCND1, CREB, GHR, GHRH, GHRHR, GNAS, SSTR2, BMP4, DRD2, FGF4, TGFA, CCNE1, HDAC2, NR3C1, SmarcA4, DKC1, MEG3, PITX2, PLAGL1, PRKCA, AKT1, AKT2, BAG1, CCNA1, CCNB1, CCNB2, CDKN1A, CDKN2A, POUO1F1, CDKN1B, PTTG, DAPK1, EGFR, LGALS3, MYO5A, NME1, RB1, COPS5, HRAS |
| Pituitary Tumors | CCNB2, CCND1, CDKN1B, HMGA2, FGFR4, PTTG1, RB1, CDKN2A |
| Acromegaly pathogenesis and treatment | GNAS, CREB, AIP, MEN1, PRKAR1A, HRAS, CCNB2, CCND1, HMGA2, FGFR4, PTTG1, RB1, CDKN1B, GADD45G, MEG3 |
| Genetic mutations in sporadic pituitary adenomas—what to screen for? | AIP, MEN1, CDKN1B, PRKAR1A, PRKACA, PRKACB, PDE11A, PDE8B, GNAS |
| Very low frequency of germline GPR101 genetic variation and no biallelic defects with AIP in a large cohort of patients with sporadic pituitary adenomas | GPR101 |
| Landscape of somatic mutations in sporadic GH-secreting pituitary adenomas | GNAS, PRKAA2, ADRBK2, ATP6V0A1, CCR10, CHRM3, OR51B4, CACNA1H, CAPN1, DMD, GRIN2B, JPH2, MAN1A1, PCDH11X, PROCA1, SLIT2, SPTA1, TESC |
| Genomic Alterations and Complex Subclonal Architecture in Sporadic GH-Secreting Pituitary Adenomas | GNAS, AIP, GPR101 |
| The Role of Epigenetic Modification in Tumorigenesis and Progression of Pituitary Adenomas: A Systematic Review of the Literature | CDKN2A, RB1, CDKN1A, DAPK1, TP73, GADD45G, FGFR2, CDH1, CASP8, RASSF1, RHBDD3, TIMP3, MGMT, THBS1, S100A10, MAGEA3, PTTG1, GNAS, NNAT, MEG3, DNMT3B, IK, HMGA2 |
| Recurrent gain-of-function USP8 mutations in Cushing’s disease | USP8 |
| Common variants at 10p12.31, 10q21.1 and 13q12.13 are associated with sporadic pituitary adenoma | NEBL, PCDH15, CDK8 |
| Lower PRDM2 expression is associated with dopamine-agonist resistance and tumor recurrence in prolactinomas | PERM1, MUCL3, DSPP, KRTAP10-3, POTEF, MUC4, MX2, PRDM2, PRG4, RP1L1 |
| Genetics of Pituitary Adenomas | GNAS, MEN1, AIP, MEN4, DICER1, SDHA, SDHB, SDHC, SDHD, AKT1, AKT2, BAG1, BMI1, BMP4, CCNA1, CCNB1, CCNB2, CCND1, CCNE1, CDKN1A, CDKN1B, CDKN2A, CDKN2B, CDKN2C, COPS5, CREB, DAPK1, DKC1, DRD2, EGFR, FGF2, FGFR1, FGFR2, FGFR4, FOLR1, GADD45B, GADD45G, GHR, GHRH, GHRHR, GNAI2, GNAS, HDAC2, HMGA1, HMGA2, HRAS, IKZF1, LAPTM4B, MAGEA3, MEG3, MERTK, NR3C1, ODC1, PIK3CA, PITX2, PLAGL1, POU1F1, PRKCA, PTTG1, PTTG1IP, RB1, SMARCA4, SSTR2, THRB, TP53, WIF1 |
| Whole-Genome Sequencing of Growth Hormone (GH)-Secreting Pituitary Adenomas | ZC3H8, ZNF438, SUPV3L1, MDGA2, C2CD3, ATPAF2, GNAS, ATAD2B, AOX1, BFSP2, SSR3, ACTN4, SUSD2, QRSL1, RUFY2, PYGM, NDUFA9, RYR1, ZNF41, PGK1, DICER1, ITPRID1, EPPK1, PTPRO, SDE2, EMC9, VPS13D, P4HA1, CACNG3, PARVB, UBR4, RHOQ, ABCB8, TMEFF1, UBAP2, PTCHD1, ARHGAP20, JAM3, VSIG4, LRP1B, FBXO15, COL24A1, PAPPA2, ARNT, NOS1AP, GPR137B, PCNXL2, GREM2, HIBADH, CD40 |
| Whole-Exome Sequencing Studies of Nonfunctioning Pituitary Adenomas | ABCA10, ASS1, CRTAC1, DOCK9, GRM7, KEL, KLHL4, MYBPH, NDRG4, NFXL1, PDGFDPOMT2, PPP3R2, RNF135, ROPN1L, SETBP1, SLC35E3, SLC5A10, SORCS1, SPHKAP, SPTBN5, TCF7L2, TOMM70A, ZAK |
| Epigenetics of pituitary tumours: an update | ACTA1, ADRA1A, ALX4, BCL9L, BIK, BMP8A, C1QTNF5, CGAS, CHST8, CNFN, COL1A2, CRIP1, DLX5, EFEMP1, EFS, ELN, EML2, ENTPD2, ERBB2, HAAO, HAS1, HDAC11, HOXB1, KCNE3, KCNQ1, TBC1D9, HHIPL1, LDHC, MT1G, NPPB, PDLIM4, PGLYRP1, PON3, RAB34, RAC2, RASSF1, RHOD, SECTM1, SIPA1, SLC5A10, VASN, SOCS1, SOCS2, ST6GALNAC6, TCF15, TFAP2E, TNFRSF10D, UBTD1, VILL, WFIKKN2 |
| Low levels of PRB3 mRNA are associated with dopamine-agonist resistance and tumor recurrence in prolactinomas | PRB3, PRG4, MUC4, DSPP, MUCL3, RP1L1, MX2, POTEF, PERM1, KRTAP10-3 |

| **Supplementary Table S6. Enrichment results for genes reported in previous studies** | | | | | |
| --- | --- | --- | --- | --- | --- |
| **Gene_set** | **Rank** | **Description** | **p_value** | **Genes** |  |
| Rare_Diseases_GeneRIF_Gene_Lists | 1 | Pituitary_cancer | 1.33E-60 | [RB1, CDKN1A, CDKN1B, THRB, PRDM2, IKZF1, NR3C1, FGF2, GHR, LGALS3, CCND1, PTTG1, CDH1, AKT1, PITX2, PRKACA, MEN1, USP8, POU1F1, MMP1, AIP, DICER1, SDHD, SDHA, SSTR2, SDHB, MMP9, PIK3CA, PRKAR1A, TP53, MEG3, HDAC2, PTTG1IP, THBS1, EGFR, GHRHR, CCNB1, PDE11A, ERBB2, DRD2, CDKN2C, CDKN2A, MGMT, HMGA1, HMGA2, IGF1, GADD45G, BMP4, GHRH, GNAS, FGFR4, GPR101, FOLR1] |  |
|  | 2 | Hypothalamic_dysfunction | 2.40E-36 | [MEG3, CDKN1B, PRDM2, NR3C1, FGF2, EGFR, GHR, LGALS3, GHRHR, CCNB1, CCND1, PTTG1, ERBB2, PRKACA, DRD2, MEN1, USP8, POU1F1, MGMT, IGFBP3, AIP, HMGA1, HMGA2, SDHD, IGF1, SDHA, SSTR2, SDHB, MMP9, GHRH, GNAS, FGFR4, GPR101, TP53, FOLR1] |  |
|  | 3 | Acromegaly | 1.65E-28 | [RB1, CDKN1B, FGF2, NPPB, SOCS2, GHR, GHRHR, NRAS, PDE11A, PTTG1, CDH1, PLAGL1, PRKACA, DRD2, POU1F1, UGT1A1, IGFBP3, MMP2, AIP, SDHD, IGF1, SSTR2, PRKAR1A, GHRH, GNAS, FGFR4, GPR101, TP53] |  |
|  | 4 | Thyroid_cancer_follicular | 3.27E-25 | [CDKN1A, CDKN1B, THRB, THBS1, EGFR, LGALS3, RASSF1, CCNB1, NRAS, CCND1, PTTG1, CDH1, ERBB2, AKT1, TIMP3, HRAS, CDKN2A, MMP2, HMGA2, PRKCA, DICER1, SDHD, IGF1, SDHB, MMP9, NME1, COPS5, PIK3CA, KRAS, TP53] |  |
|  | 5 | Adrenal_cancer | 5.05E-23 | [CDKN1A, CDKN1B, NR3C1, EGFR, PDE11A, CCND1, PTTG1, ERBB2, AKT1, PDE8B, PRKACA, DRD2, HRAS, MEN1, CDKN2A, SDHC, SDHD, IGF1, SDHA, SSTR2, SDHB, PIK3CA, PRKAR1A, GNAS, KRAS, TP53] |  |
| GO_Biological_Process_2018 | 1 | regulation of cell proliferation (GO:0042127) | 9.22E-13 | [CD40, CDKN1A, HDAC2, CDKN1B, TGFA, FGF2, ADRA1A, THBS1, EGFR, FGF4, GHRHR, AKT2, AKT1, CAPN1, DRD2, HRAS, MEN1, TCF7L2, CDKN2B, POU1F1, CDKN2C, TFAP2E, CDKN2A, TESC, IGFBP3, HMGA1, IGF1, SSTR2, NME1, TNFRSF10D, BMP4, GHRH, KRAS, FGFR4, FOLR2, TP53, FGFR2, FGFR1] |  |
|  | 2 | positive regulation of phosphorylation (GO:0042327) | 5.32E-11 | [CD40, PRKAA2, ARNT, IGF1, MERTK, MMP9, THBS1, EGFR, BMP4, CCND3, CCND1, AKT2, ERBB2, AKT1, KRAS, FGFR4, HRAS, FGFR2, FGFR1] |  |
|  | 3 | positive regulation of cellular process (GO:0048522) | 8.01E-11 | [HDAC2, CDKN1B, TGFA, FGF2, THBS1, EGFR, FGF4, GHRHR, CCND1, AKT2, ERBB2, AKT1, CAPN1, HRAS, HMGA2, ACTN4, DICER1, IGF1, SUPV3L1, BMP4, MMP14, KCNQ1, GHRH, NOS1AP, KRAS, FGFR4, FOLR2, FGFR2, FGFR1] |  |
|  | 4 | negative regulation of apoptotic process (GO:0043066) | 8.21E-11 | [HDAC2, PRKAA2, THBS1, EGFR, SOCS2, AKT2, BAG1, ERBB2, AKT1, MAGEA3, HTATIP2, MGMT, HMGA2, PRKCA, IGF1, MERTK, MMP9, TNFRSF10D, SUPV3L1, BMP4, COPS5, PIK3CA, FGFR4, TP53, CD44, FGFR2, TP73, FGFR1] |  |
|  | 5 | regulation of apoptotic process (GO:0042981) | 1.02E-09 | [CD40, HDAC2, PRKAA2, THBS1, EGFR, SOCS2, AKT2, BAG1, ERBB2, AKT1, SLIT2, HTATIP2, GADD45B, MGMT, DAPK1, IGFBP3, BMP8A, HMGA2, ACTN4, IGF1, MERTK, MMP9, GADD45G, NME1, TNFRSF10D, SUPV3L1, BMP4, COPS5, NOS1AP, FGFR4, TP53, CD44, FGFR2, TP73, FGFR1] |  |
| GO_Cellular_Component_2018 | 1 | cytoskeleton (GO:0005856) | 2.38E-05 | [DAPK1, ACTN4, KLHL4, PARVB, EML2, SPTA1, CENPE, CCNA1, CCNB2, ACTA1, RASSF1, MMP14, CASP8, CDH1, RAC2, AKT1, EPPK1, VILL, PDLIM4, RHOQ] |  |
|  | 2 | anchored component of external side of plasma membrane (GO:0031362) | 1.64E-03 | [FOLR3, FOLR2, FOLR1] |  |
|  | 3 | actin cytoskeleton (GO:0015629) | 2.08E-03 | [SPTA1, ACTA1, CDH1, DAPK1, MYBPH, RAC2, MYO5A, ACTN4, PARVB, VILL, RHOQ] |  |
|  | 4 | intrinsic component of external side of plasma membrane (GO:0031233) | 2.97E-03 | [FOLR3, FOLR2, FOLR1] |  |
|  | 5 | RNA polymerase II transcription factor complex (GO:0090575) | 3.71E-03 | [RB1, THRB, HMGA1, ARNT, GTF2H1, TP53, BCL9L] |  |
| GO_Molecular_Function_2018 | 1 | protein kinase binding (GO:0019901) | 2.98E-09 | [CDKN1A, CDKN1B, NR3C1, EGFR, GHR, CCND3, CCNB1, SOCS1, CCND1, ERBB2, RAC2, PRKACA, TCF7L2, CDKN2B, CDKN2C, CDKN2A, MERTK, RHOD, PRKAR1A, KCNQ1, FGFR4, TP53, RHOQ, FGFR2, TP73, FGFR1] |  |
|  | 2 | cyclin-dependent protein serine/threonine kinase inhibitor activity (GO:0004861) | 9.56E-08 | [CDKN1A, CDKN2B, CDKN2C, CDKN1B, CDKN2A] |  |
|  | 3 | kinase binding (GO:0019900) | 2.22E-07 | [RB1, TCF7L2, CDKN2B, CDKN1A, CDKN2C, CDKN1B, CDKN2A, NR3C1, RHOD, AURKB, GHR, LAPTM4B, CCND3, CCNB1, SOCS1, CCND1, RAC2, PRKACA, TP53, RHOQ, TP73] |  |
|  | 4 | cyclin-dependent protein serine/threonine kinase regulator activity (GO:0016538) | 1.54E-06 | [CDKN1A, CCNB1, CDKN2B, CDKN1B, CDKN2C, CDKN2A] |  |
|  | 5 | protein serine/threonine kinase inhibitor activity (GO:0030291) | 2.91E-06 | [CDKN1A, CDKN2B, CDKN1B, CDKN2C, CDKN2A, PRKAR1A] |  |

| **Supplementary Table S7. ANNOVAR annotation results. Both WES and targeted resequencing data were searched in COSMICv70 and ClinVar database.** | | | | | | | | | | | | | | | |  |  |  |  |
| --- | --- | --- | --- | --- | --- | --- | --- | --- | --- | --- | --- | --- | --- | --- | --- | --- | --- | --- | --- |
| **Chr** | **Start** | **End** | **Ref** | **Alt** | **Func** | **Gene** | **ExonicFunc** | **AAChange** | **cosmic70** | **CLNALLELEID** | **CLNDN** | **CLNDISDB** | **CLNREVSTAT** | **CLNSIG** |  |  |  |  |  |
| 5 | 89939662 | 89939662 | C | T | exonic | ADGRV1 | nonsynonymous SNV | ADGRV1:NM_032119:exon14:c.C2596T:p.R866W | . | 490842 | Usher_syndrome\x2c_type_2C\|not_specified\|not_provided | MONDO:MONDO:0011558\x2cMedGen:C2931213\x2cOMIM:605472\|MedGen:CN169374\|MedGen:CN517202 | criteria_provided\x2c_multiple_submitters\x2c_no_conflicts | Uncertain_significance |  |  |  |  |  |
| 5 | 89938542 | 89938542 | A | C | exonic | ADGRV1 | nonsynonymous SNV | ADGRV1:NM_032119:exon12:c.A2330C:p.E777A | . | 894871 | Usher_syndrome\x2c_type_2C\|not_provided | MONDO:MONDO:0011558\x2cMedGen:C2931213\x2cOMIM:605472\|MedGen:CN517202 | criteria_provided\x2c_multiple_submitters\x2c_no_conflicts | Uncertain_significance |  |  |  |  |  |
| 7 | 21658769 | 21658769 | C | T | exonic | DNAH11 | nonsynonymous SNV | DNAH11:NM_001277115:exon24:c.C4306T:p.R1436W | ID=COSM1622696,COSM3663091;OCCURENCE=2(liver) | 305716 | Primary_ciliary_dyskinesia | Human_Phenotype_Ontology:HP:0012265\x2cMONDO:MONDO:0016575\x2cMedGen:C4551720\x2cOMIM:PS244400\x2cOrphanet:ORPHA244 | criteria_provided\x2c_conflicting_interpretations | Conflicting_interpretations_of_pathogenicity |  |  |  |  |  |
| 7 | 21894036 | 21894036 | T | C | exonic | DNAH11 | nonsynonymous SNV | DNAH11:NM_001277115:exon68:c.T11165C:p.L3722P | . | 395852 | Primary_ciliary_dyskinesia | Human_Phenotype_Ontology:HP:0012265\x2cMONDO:MONDO:0016575\x2cMedGen:C4551720\x2cOMIM:PS244400\x2cOrphanet:ORPHA244 | criteria_provided\x2c_single_submitter | Likely_pathogenic |  |  |  |  |  |
| 7 | 21630947 | 21630947 | G | C | exonic | DNAH11 | nonsynonymous SNV | DNAH11:NM_001277115:exon14:c.G2419C:p.D807H | . | 310507 | Primary_ciliary_dyskinesia | Human_Phenotype_Ontology:HP:0012265\x2cMONDO:MONDO:0016575\x2cMedGen:C4551720\x2cOMIM:PS244400\x2cOrphanet:ORPHA244 | criteria_provided\x2c_conflicting_interpretations | Conflicting_interpretations_of_pathogenicity |  |  |  |  |  |
| 7 | 111382130 | 111382130 | C | T | exonic | DOCK4 | nonsynonymous SNV | DOCK4:NM_014705:exon44:c.G4709A:p.R1570K,DOCK4:NM_001363540:exon45:c.G4736A:p.R1579K | . | 735887 | not_provided | MedGen:CN517202 | criteria_provided\x2c_single_submitter | Benign |  |  |  |  |  |
| 3 | 129305540 | 129305540 | T | C | exonic | PLXND1 | nonsynonymous SNV | PLXND1:NM_015103:exon3:c.A1511G:p.Q504R | . | 733789 | not_provided | MedGen:CN517202 | criteria_provided\x2c_single_submitter | Benign |  |  |  |  |  |
| 10 | 16979723 | 16979723 | C | G | exonic | CUBN | nonsynonymous SNV | CUBN:NM_001081:exon39:c.G5794C:p.E1932Q | . | 865690 | Imerslund-GrÃ¤sbeck_syndrome_1 | MONDO:MONDO:0100156\x2cMedGen:C4016819\x2cOMIM:261100 | criteria_provided\x2c_single_submitter | Uncertain_significance |  |  |  |  |  |
| 1 | 175096130 | 175096130 | C | T | exonic | TNN | nonsynonymous SNV | TNN:NM_022093:exon13:c.C2954T:p.T985M | ID=COSM899910;OCCURENCE=1(endometrium) | . | . | . | . | . |  |  |  |  |  |
| 14 | 23944783 | 23944783 | G | A | exonic | NGDN | nonsynonymous SNV | NGDN:NM_001042635:exon5:c.G299A:p.R100H,NGDN:NM_015514:exon5:c.G299A:p.R100H | ID=COSM552651,COSM552652;OCCURENCE=1(lung) | . | . | . | . | . |  |  |  |  |  |
| 11 | 123813764 | 123813764 | C | T | exonic | OR6T1 | nonsynonymous SNV | OR6T1:NM_001005187:exon1:c.G782A:p.R261H | ID=COSM202900;OCCURENCE=1(large_intestine),2(urinary_tract) | . | . | . | . | . |  |  |  |  |  |
| 12 | 123950638 | 123950638 | G | A | exonic | SNRNP35 | nonsynonymous SNV | SNRNP35:NM_022717:exon2:c.G551A:p.R184Q,SNRNP35:NM_180699:exon2:c.G566A:p.R189Q | ID=COSM1242871;OCCURENCE=1(oesophagus) | . | . | . | . | . |  |  |  |  |  |
| 2 | 136673874 | 136673874 | G | A | exonic | DARS1 | nonsynonymous SNV | DARS1:NM_001293312:exon10:c.C728T:p.T243I,DARS1:NM_001349:exon11:c.C1028T:p.T343I | . | . | . | . | . | . |  |  |  |  |  |
| 14 | 23944471 | 23944471 | C | G | exonic | NGDN | nonsynonymous SNV | NGDN:NM_001042635:exon4:c.C236G:p.S79C,NGDN:NM_015514:exon4:c.C236G:p.S79C | . | . | . | . | . | . |  |  |  |  |  |
| 19 | 50944154 | 50944156 | AGA | - | exonic | MYBPC2 | nonframeshift deletion | MYBPC2:NM_004533:exon8:c.590_592del:p.K203del | . | . | . | . | . | . |  |  |  |  |  |
| 6 | 57372291 | 57372291 | A | C | exonic | PRIM2 | unknown | UNKNOWN | . | . | . | . | . | . |  |  |  |  |  |
| 19 | 50962554 | 50962554 | C | T | exonic | MYBPC2 | nonsynonymous SNV | MYBPC2:NM_004533:exon23:c.C2782T:p.R928C | . | . | . | . | . | . |  |  |  |  |  |
| 5 | 66459810 | 66459810 | C | A | exonic | MAST4 | nonsynonymous SNV | MAST4:NM_001290227:exon26:c.C4020A:p.S1340R,MAST4:NM_001297651:exon26:c.C4221A:p.S1407R,MAST4:NM_001290226:exon27:c.C4185A:p.S1395R,MAST4:NM_015183:exon28:c.C4236A:p.S1412R,MAST4:NM_001164664:exon29:c.C4803A:p.S1601R | . | . | . | . | . | . |  |  |  |  |  |
| 3 | 129290559 | 129290559 | G | A | exonic | PLXND1 | nonsynonymous SNV | PLXND1:NM_015103:exon16:c.C3206T:p.P1069L | . | . | . | . | . | . |  |  |  |  |  |
| 10 | 73051306 | 73051306 | C | G | exonic | UNC5B | nonsynonymous SNV | UNC5B:NM_001244889:exon9:c.C1379G:p.S460C,UNC5B:NM_170744:exon10:c.C1412G:p.S471C | . | . | . | . | . | . |  |  |  |  |  |
| 7 | 111449410 | 111449410 | C | G | exonic | DOCK4 | nonsynonymous SNV | DOCK4:NM_001363540:exon29:c.G3054C:p.L1018F,DOCK4:NM_014705:exon29:c.G3054C:p.L1018F | . | . | . | . | . | . |  |  |  |  |  |
| 11 | 1024043 | 1024043 | C | G | exonic | MUC6 | nonsynonymous SNV | MUC6:NM_005961:exon25:c.G3286C:p.G1096R | . | . | . | . | . | . |  |  |  |  |  |
| 3 | 165547863 | 165547863 | G | A | exonic | BCHE | nonsynonymous SNV | BCHE:NM_000055:exon2:c.C959T:p.P320L | . | . | . | . | . | . |  |  |  |  |  |
| 3 | 165491266 | 165491266 | C | A | exonic | BCHE | nonsynonymous SNV | BCHE:NM_000055:exon4:c.G1713T:p.W571C | . | . | . | . | . | . |  |  |  |  |  |
| 14 | 103414073 | 103414073 | A | C | exonic | CDC42BPB | nonsynonymous SNV | CDC42BPB:NM_006035:exon27:c.T3516G:p.I1172M | . | . | . | . | . | . |  |  |  |  |  |
| 1 | 9791951 | 9791951 | T | G | exonic | CLSTN1 | nonsynonymous SNV | CLSTN1:NM_001302883:exon16:c.A2374C:p.N792H,CLSTN1:NM_014944:exon16:c.A2401C:p.N801H,CLSTN1:NM_001009566:exon17:c.A2431C:p.N811H | . | . | . | . | . | . |  |  |  |  |  |
| 6 | 57498980 | 57498980 | T | G | exonic | PRIM2 | unknown | UNKNOWN | . | . | . | . | . | . |  |  |  |  |  |
| 1 | 175049397 | 175049397 | T | C | exonic | TNN | nonsynonymous SNV | TNN:NM_022093:exon4:c.T883C:p.Y295H | . | . | . | . | . | . |  |  |  |  |  |
| 6 | 57512696 | 57512696 | T | C | exonic | PRIM2 | unknown | UNKNOWN | . | . | . | . | . | . |  |  |  |  |  |
| 5 | 66391477 | 66391477 | A | T | exonic | MAST4 | nonsynonymous SNV | MAST4:NM_001297651:exon4:c.A304T:p.T102S,MAST4:NM_001290226:exon5:c.A268T:p.T90S,MAST4:NM_001290227:exon5:c.A304T:p.T102S,MAST4:NM_015183:exon6:c.A319T:p.T107S,MAST4:NM_001164664:exon7:c.A886T:p.T296S | . | . | . | . | . | . |  |  |  |  |  |
| 10 | 73046626 | 73046626 | G | A | exonic | UNC5B | nonsynonymous SNV | UNC5B:NM_001244889:exon5:c.G733A:p.V245M,UNC5B:NM_170744:exon5:c.G733A:p.V245M | . | . | . | . | . | . |  |  |  |  |  |
| 1 | 175046736 | 175046736 | A | - | exonic | TNN | frameshift deletion | TNN:NM_022093:exon2:c.182delA:p.D61Afs*52 | . | . | . | . | . | . |  |  |  |  |  |
| 10 | 16967396 | 16967396 | G | A | exonic | CUBN | nonsynonymous SNV | CUBN:NM_001081:exon43:c.C6490T:p.P2164S | . | . | . | . | . | . |  |  |  |  |  |
| 1 | 53972347 | 53972347 | T | C | exonic | GLIS1 | nonsynonymous SNV | GLIS1:NM_147193:exon10:c.A1808G:p.N603S,GLIS1:NM_001367484:exon11:c.A2333G:p.N778S | . | . | . | . | . | . |  |  |  |  |  |
| 5 | 66055618 | 66055618 | C | A | exonic | MAST4 | nonsynonymous SNV | MAST4:NM_001164664:exon2:c.C445A:p.L149M,MAST4:NM_001290228:exon2:c.C445A:p.L149M,MAST4:NM_198828:exon2:c.C445A:p.L149M | . | . | . | . | . | . |  |  |  |  |  |
| 8 | 139838919 | 139838919 | G | T | exonic | COL22A1 | nonsynonymous SNV | COL22A1:NM_152888:exon6:c.C951A:p.D317E | . | . | . | . | . | . |  |  |  |  |  |
| 8 | 139845364 | 139845364 | T | C | exonic | COL22A1 | nonsynonymous SNV | COL22A1:NM_152888:exon5:c.A763G:p.K255E | . | . | . | . | . | . |  |  |  |  |  |
| 10 | 16870839 | 16870839 | C | G | exonic | CUBN | nonsynonymous SNV | CUBN:NM_001081:exon66:c.G10729C:p.A3577P | . | . | . | . | . | . |  |  |  |  |  |
| 2 | 136701012 | 136701012 | A | C | exonic | DARS1 | nonsynonymous SNV | DARS1:NM_001293312:exon4:c.T59G:p.V20G,DARS1:NM_001349:exon5:c.T359G:p.V120G | . | . | . | . | . | . |  |  |  |  |  |
| 7 | 21726866 | 21726866 | A | G | exonic | DNAH11 | nonsynonymous SNV | DNAH11:NM_001277115:exon33:c.A5771G:p.D1924G | . | . | . | . | . | . |  |  |  |  |  |
| 7 | 111379484 | 111379484 | T | A | exonic | DOCK4 | nonsynonymous SNV | DOCK4:NM_014705:exon47:c.A5063T:p.N1688I,DOCK4:NM_001363540:exon48:c.A5090T:p.N1697I | . | . | . | . | . | . |  |  |  |  |  |
| 3 | 10123043 | 10123043 | A | G | exonic | FANCD2 | nonsynonymous SNV | FANCD2:NM_001374253:exon31:c.A3008G:p.E1003G,FANCD2:NM_001018115:exon32:c.A3119G:p.E1040G,FANCD2:NM_001319984:exon32:c.A3119G:p.E1040G,FANCD2:NM_001374254:exon32:c.A3119G:p.E1040G,FANCD2:NM_033084:exon32:c.A3119G:p.E1040G | . | . | . | . | . | . |  |  |  |  |  |
| 5 | 90449105 | 90449105 | G | C | exonic | ADGRV1 | nonsynonymous SNV | ADGRV1:NM_032119:exon89:c.G18692C:p.S6231T | . | . | . | . | . | . |  |  |  |  |  |
| 5 | 66430459 | 66430459 | G | A | exonic | MAST4 | nonsynonymous SNV | MAST4:NM_001297651:exon15:c.G1753A:p.G585R,MAST4:NM_001290226:exon16:c.G1717A:p.G573R,MAST4:NM_001290227:exon16:c.G1753A:p.G585R,MAST4:NM_015183:exon17:c.G1768A:p.G590R,MAST4:NM_001164664:exon18:c.G2335A:p.G779R | . | . | . | . | . | . |  |  |  |  |  |
| 3 | 15062322 | 15062322 | A | G | exonic | NR2C2 | nonsynonymous SNV | NR2C2:NM_001291694:exon5:c.A439G:p.K147E,NR2C2:NM_003298:exon6:c.A496G:p.K166E | . | . | . | . | . | . |  |  |  |  |  |
| 3 | 129286570 | 129286570 | T | C | exonic | PLXND1 | nonsynonymous SNV | PLXND1:NM_015103:exon21:c.A3944G:p.E1315G | . | . | . | . | . | . |  |  |  |  |  |
| 17 | 21319860 | 21319860 | C | A | exonic | KCNJ12;KCNJ18 | nonsynonymous SNV | KCNJ18:NM_001194958:exon3:c.C1206A:p.D402E,KCNJ12:NM_021012:exon3:c.C1206A:p.D402E | . | . | . | . | . | . |  |  |  |  |  |
| 17 | 27283235 | 27283235 | C | G | exonic | SEZ6 | nonsynonymous SNV | SEZ6:NM_001098635:exon16:c.G2894C:p.R965P,SEZ6:NM_001290202:exon16:c.G2519C:p.R840P,SEZ6:NM_178860:exon16:c.G2894C:p.R965P | . | . | . | . | . | . |  |  |  |  |  |
| 6 | 57398226 | 57398226 | T | A | exonic | PRIM2 | unknown | UNKNOWN | . | . | . | . | . | . |  |  |  |  |  |
| 10 | 73050759 | 73050759 | T | G | exonic | UNC5B | nonsynonymous SNV | UNC5B:NM_001244889:exon8:c.T1154G:p.V385G,UNC5B:NM_170744:exon9:c.T1187G:p.V396G | . | . | . | . | . | . |  |  |  |  |  |
| 1 | 175116071 | 175116071 | T | G | exonic | TNN | nonsynonymous SNV | TNN:NM_022093:exon19:c.T3764G:p.V1255G | . | . | . | . | . | . |  |  |  |  |  |
| 11 | 1018341 | 1018341 | G | A | exonic | MUC6 | nonsynonymous SNV | MUC6:NM_005961:exon31:c.C4460T:p.P1487L | . | . | . | . | . | . |  |  |  |  |  |
| 14 | 64066398 | 64066398 | G | C | exonic | WDR89 | nonsynonymous SNV | WDR89:NM_001258272:exon3:c.C263G:p.A88G,WDR89:NM_080666:exon3:c.C263G:p.A88G,WDR89:NM_001008726:exon4:c.C263G:p.A88G | . | . | . | . | . | . |  |  |  |  |  |
| 14 | 64066402 | 64066402 | A | G | exonic | WDR89 | nonsynonymous SNV | WDR89:NM_001258272:exon3:c.T259C:p.S87P,WDR89:NM_080666:exon3:c.T259C:p.S87P,WDR89:NM_001008726:exon4:c.T259C:p.S87P | . | . | . | . | . | . |  |  |  |  |  |
| 19 | 50963404 | 50963404 | T | G | exonic | MYBPC2 | nonsynonymous SNV | MYBPC2:NM_004533:exon24:c.T2899G:p.Y967D | . | . | . | . | . | . |  |  |  |  |  |
| 14 | 64066363 | 64066363 | G | A | exonic | WDR89 | stopgain | WDR89:NM_001258272:exon3:c.C298T:p.R100X,WDR89:NM_080666:exon3:c.C298T:p.R100X,WDR89:NM_001008726:exon4:c.C298T:p.R100X | . | . | . | . | . | . |  |  |  |  |  |
| 14 | 64066367 | 64066367 | A | T | exonic | WDR89 | nonsynonymous SNV | WDR89:NM_001258272:exon3:c.T294A:p.D98E,WDR89:NM_080666:exon3:c.T294A:p.D98E,WDR89:NM_001008726:exon4:c.T294A:p.D98E | . | . | . | . | . | . |  |  |  |  |  |
| 6 | 32552026 | 32552026 | C | T | exonic | HLA-DRB1 | nonsynonymous SNV | HLA-DRB1:NM_002124:exon2:c.G230A:p.R77Q | . | . | . | . | . | . |  |  |  |  |  |
| 14 | 105399147 | 105399147 | T | G | exonic | PLD4 | nonsynonymous SNV | PLD4:NM_001308174:exon11:c.T1388G:p.V463G,PLD4:NM_138790:exon11:c.T1367G:p.V456G | . | . | . | . | . | . |  |  |  |  |  |
| 14 | 64066352 | 64066352 | T | A | exonic | WDR89 | nonsynonymous SNV | WDR89:NM_001258272:exon3:c.A309T:p.R103S,WDR89:NM_080666:exon3:c.A309T:p.R103S,WDR89:NM_001008726:exon4:c.A309T:p.R103S | . | . | . | . | . | . |  |  |  |  |  |
| 6 | 32549613 | 32549613 | G | A | exonic | HLA-DRB1 | stopgain | HLA-DRB1:NM_002124:exon3:c.C373T:p.Q125X | . | . | . | . | . | . |  |  |  |  |  |
| 13 | 25670767 | 25670767 | A | G | exonic | PABPC3 | nonsynonymous SNV | PABPC3:NM_030979:exon1:c.A431G:p.H144R | . | . | . | . | . | . |  |  |  |  |  |
| 13 | 25670797 | 25670797 | C | G | exonic | PABPC3 | nonsynonymous SNV | PABPC3:NM_030979:exon1:c.C461G:p.A154G | . | . | . | . | . | . |  |  |  |  |  |
| 14 | 64066326 | 64066326 | C | T | exonic | WDR89 | nonsynonymous SNV | WDR89:NM_001258272:exon3:c.G335A:p.G112D,WDR89:NM_080666:exon3:c.G335A:p.G112D,WDR89:NM_001008726:exon4:c.G335A:p.G112D | . | . | . | . | . | . |  |  |  |  |  |
| 17 | 32483479 | 32483479 | T | G | exonic | ASIC2 | nonsynonymous SNV | ASIC2:NM_001094:exon1:c.A73C:p.T25P | . | . | . | . | . | . |  |  |  |  |  |
| 2 | 70524437 | 70524437 | A | G | exonic | FAM136A | nonsynonymous SNV | FAM136A:NM_001329752:exon3:c.T722C:p.L241S,FAM136A:NM_001329753:exon3:c.T656C:p.L219S,FAM136A:NM_001329755:exon3:c.T308C:p.L103S,FAM136A:NM_032822:exon3:c.T401C:p.L134S,FAM136A:NM_001329757:exon4:c.T308C:p.L103S,FAM136A:NM_001329758:exon4:c.T308C:p.L103S | . | . | . | . | . | . |  |  |  |  |  |
| 20 | 44171347 | 44171347 | T | G | exonic | EPPIN;EPPIN-WFDC6 | nonsynonymous SNV | EPPIN-WFDC6:NM_001198986:exon3:c.A383C:p.K128T,EPPIN:NM_020398:exon3:c.A383C:p.K128T | . | . | . | . | . | . |  |  |  |  |  |
| 8 | 27394350 | 27394350 | G | T | exonic | EPHX2 | nonsynonymous SNV | EPHX2:NM_001256483:exon12:c.G1022T:p.S341I,EPHX2:NM_001256482:exon13:c.G1061T:p.S354I,EPHX2:NM_001256484:exon13:c.G1061T:p.S354I,EPHX2:NM_001979:exon13:c.G1220T:p.S407I | . | . | . | . | . | . |  |  |  |  |  |
| 13 | 25670712 | 25670712 | C | G | exonic | PABPC3 | nonsynonymous SNV | PABPC3:NM_030979:exon1:c.C376G:p.L126V | . | . | . | . | . | . |  |  |  |  |  |
| 1 | 53975587 | 53975587 | A | G | exonic | GLIS1 | nonsynonymous SNV | GLIS1:NM_147193:exon8:c.T1472C:p.L491P,GLIS1:NM_001367484:exon9:c.T1997C:p.L666P | . | . | . | . | . | . |  |  |  |  |  |
| 1 | 9795042 | 9795042 | C | T | exonic | CLSTN1 | nonsynonymous SNV | CLSTN1:NM_001302883:exon13:c.G2017A:p.V673M,CLSTN1:NM_014944:exon13:c.G2044A:p.V682M,CLSTN1:NM_001009566:exon14:c.G2074A:p.V692M | . | . | . | . | . | . |  |  |  |  |  |
| 11 | 123813857 | 123813857 | G | C | exonic | OR6T1 | nonsynonymous SNV | OR6T1:NM_001005187:exon1:c.C689G:p.T230R | . | . | . | . | . | . |  |  |  |  |  |
| 11 | 1888065 | 1888065 | G | A | exonic | LSP1 | nonsynonymous SNV | LSP1:NM_001242932:exon2:c.G361A:p.G121S | . | . | . | . | . | . |  |  |  |  |  |
| 13 | 25670730 | 25670730 | T | G | exonic | PABPC3 | nonsynonymous SNV | PABPC3:NM_030979:exon1:c.T394G:p.C132G | . | . | . | . | . | . |  |  |  |  |  |
| 14 | 101347290 | 101347290 | C | G | exonic | RTL1 | nonsynonymous SNV | RTL1:NM_001134888:exon1:c.G3836C:p.R1279P | . | . | . | . | . | . |  |  |  |  |  |
| 14 | 103414073 | 103414073 | A | C | exonic | CDC42BPB | nonsynonymous SNV | CDC42BPB:NM_006035:exon27:c.T3516G:p.I1172M | . | . | . | . | . | . |  |  |  |  |  |
| 16 | 75646247 | 75646247 | C | T | exonic | ADAT1 | nonsynonymous SNV | ADAT1:NM_001324452:exon5:c.G676A:p.G226R,ADAT1:NM_001324453:exon5:c.G676A:p.G226R,ADAT1:NM_001324445:exon6:c.G937A:p.G313R,ADAT1:NM_001324449:exon6:c.G937A:p.G313R,ADAT1:NM_001324451:exon6:c.G490A:p.G164R,ADAT1:NM_001324444:exon7:c.G490A:p.G164R,ADAT1:NM_001324446:exon7:c.G490A:p.G164R,ADAT1:NM_001324448:exon7:c.G937A:p.G313R,ADAT1:NM_001324450:exon7:c.G490A:p.G164R,ADAT1:NM_012091:exon7:c.G937A:p.G313R | . | . | . | . | . | . |  |  |  |  |  |
| 19 | 42596335 | 42596335 | A | G | exonic | POU2F2 | nonsynonymous SNV | POU2F2:NM_001207025:exon13:c.T1286C:p.L429P,POU2F2:NM_001207026:exon13:c.T1286C:p.L429P,POU2F2:NM_002698:exon13:c.T1238C:p.L413P | . | . | . | . | . | . |  |  |  |  |  |
| 19 | 9324835 | 9324835 | C | T | exonic | OR7D4 | nonsynonymous SNV | OR7D4:NM_001005191:exon1:c.G679A:p.G227R | . | . | . | . | . | . |  |  |  |  |  |
| 2 | 136681989 | 136681989 | A | T | exonic | DARS1 | nonsynonymous SNV | DARS1:NM_001293312:exon7:c.T344A:p.F115Y,DARS1:NM_001349:exon8:c.T644A:p.F215Y | . | . | . | . | . | . |  |  |  |  |  |
| 2 | 70524444 | 70524444 | C | G | exonic | FAM136A | nonsynonymous SNV | FAM136A:NM_001329752:exon3:c.G715C:p.A239P,FAM136A:NM_001329753:exon3:c.G649C:p.A217P,FAM136A:NM_001329755:exon3:c.G301C:p.A101P,FAM136A:NM_032822:exon3:c.G394C:p.A132P,FAM136A:NM_001329757:exon4:c.G301C:p.A101P,FAM136A:NM_001329758:exon4:c.G301C:p.A101P | . | . | . | . | . | . |  |  |  |  |  |
| 22 | 17590313 | 17590313 | C | T | exonic | IL17RA | nonsynonymous SNV | IL17RA:NM_001289905:exon12:c.C2102T:p.A701V,IL17RA:NM_014339:exon13:c.C2204T:p.A735V | . | . | . | . | . | . |  |  |  |  |  |
| 3 | 142268442 | 142268442 | A | G | exonic | ATR | nonsynonymous SNV | ATR:NM_001354579:exon14:c.T2858C:p.L953S,ATR:NM_001184:exon15:c.T3050C:p.L1017S | . | . | . | . | . | . |  |  |  |  |  |
| 3 | 15079612 | 15079612 | C | T | exonic | NR2C2 | nonsynonymous SNV | NR2C2:NM_001291694:exon12:c.C1478T:p.A493V,NR2C2:NM_003298:exon13:c.C1535T:p.A512V | . | . | . | . | . | . |  |  |  |  |  |
| 3 | 165491266 | 165491266 | C | A | exonic | BCHE | nonsynonymous SNV | BCHE:NM_000055:exon4:c.G1713T:p.W571C | . | . | . | . | . | . |  |  |  |  |  |
| 3 | 41291011 | 41291011 | G | C | exonic | ULK4 | nonsynonymous SNV | ULK4:NM_001322501:exon35:c.C2827G:p.R943G,ULK4:NM_017886:exon36:c.C3733G:p.R1245G | . | . | . | . | . | . |  |  |  |  |  |
| 4 | 164050219 | 164050219 | G | A | exonic | NAF1 | nonsynonymous SNV | NAF1:NM_138386:exon8:c.C1315T:p.P439S | . | . | . | . | . | . |  |  |  |  |  |
| 5 | 101811464 | 101811464 | G | T | exonic | SLCO6A1 | nonsynonymous SNV | SLCO6A1:NM_001289004:exon3:c.C650A:p.A217D,SLCO6A1:NM_001308014:exon3:c.C650A:p.A217D,SLCO6A1:NM_001289002:exon4:c.C836A:p.A279D,SLCO6A1:NM_173488:exon4:c.C836A:p.A279D | . | . | . | . | . | . |  |  |  |  |  |
| 5 | 101811465 | 101811465 | C | T | exonic | SLCO6A1 | nonsynonymous SNV | SLCO6A1:NM_001289004:exon3:c.G649A:p.A217T,SLCO6A1:NM_001308014:exon3:c.G649A:p.A217T,SLCO6A1:NM_001289002:exon4:c.G835A:p.A279T,SLCO6A1:NM_173488:exon4:c.G835A:p.A279T | . | . | . | . | . | . |  |  |  |  |  |
| 5 | 66055618 | 66055618 | C | A | exonic | MAST4 | nonsynonymous SNV | MAST4:NM_001164664:exon2:c.C445A:p.L149M,MAST4:NM_001290228:exon2:c.C445A:p.L149M,MAST4:NM_198828:exon2:c.C445A:p.L149M | . | . | . | . | . | . |  |  |  |  |  |
| 6 | 121604966 | 121604966 | G | A | exonic | TBC1D32 | nonsynonymous SNV | TBC1D32:NM_001367760:exon13:c.C1463T:p.S488L,TBC1D32:NM_152730:exon13:c.C1463T:p.S488L,TBC1D32:NM_001367759:exon14:c.C1463T:p.S488L | . | . | . | . | . | . |  |  |  |  |  |
| 6 | 32485520 | 32485520 | C | T | exonic | HLA-DRB5 | nonsynonymous SNV | HLA-DRB5:NM_002125:exon6:c.G797A:p.S266N | . | . | . | . | . | . |  |  |  |  |  |
| 6 | 74210324 | 74210324 | G | A | exonic | MTO1 | nonsynonymous SNV | MTO1:NM_012123:exon12:c.G1945A:p.V649I,MTO1:NM_001123226:exon13:c.G2065A:p.V689I,MTO1:NM_133645:exon13:c.G2020A:p.V674I | . | . | . | . | . | . |  |  |  |  |  |
| 7 | 136699682 | 136699682 | G | T | exonic | CHRM2 | nonsynonymous SNV | CHRM2:NM_001006629:exon2:c.G70T:p.V24L,CHRM2:NM_001006627:exon3:c.G70T:p.V24L,CHRM2:NM_001006628:exon3:c.G70T:p.V24L,CHRM2:NM_001006632:exon3:c.G70T:p.V24L,CHRM2:NM_000739:exon4:c.G70T:p.V24L,CHRM2:NM_001006630:exon4:c.G70T:p.V24L,CHRM2:NM_001006631:exon4:c.G70T:p.V24L,CHRM2:NM_001006626:exon5:c.G70T:p.V24L | . | . | . | . | . | . |  |  |  |  |  |
| 7 | 21598465 | 21598465 | T | C | exonic | DNAH11 | nonsynonymous SNV | DNAH11:NM_001277115:exon3:c.T541C:p.S181P | . | . | . | . | . | . |  |  |  |  |  |
| 7 | 23724237 | 23724237 | C | G | exonic | FAM221A | nonsynonymous SNV | FAM221A:NM_001127364:exon2:c.C185G:p.T62R,FAM221A:NM_199136:exon2:c.C185G:p.T62R | . | . | . | . | . | . |  |  |  |  |  |
| 7 | 30185850 | 30185850 | A | G | exonic | MTURN | nonsynonymous SNV | MTURN:NM_152793:exon2:c.A220G:p.I74V | . | . | . | . | . | . |  |  |  |  |  |
| 7 | 99269452 | 99269452 | A | T | exonic | CYP3A5 | nonsynonymous SNV | CYP3A5:NM_001190484:exon5:c.T368A:p.M123K | . | . | . | . | . | . |  |  |  |  |  |
| 8 | 139727938 | 139727938 | T | C | exonic | COL22A1 | nonsynonymous SNV | COL22A1:NM_152888:exon30:c.A2504G:p.D835G | . | . | . | . | . | . |  |  |  |  |  |
| 9 | 136342228 | 136342228 | C | G | exonic | SLC2A6 | nonsynonymous SNV | SLC2A6:NM_001145099:exon3:c.G391C:p.G131R,SLC2A6:NM_017585:exon3:c.G391C:p.G131R | . | . | . | . | . | . |  |  |  |  |  |

| **Supplementary Table S8. Primers used in this study** | | |
| --- | --- | --- |
| **Name of primer** | **Sequence** | **Target locus (hg19 coordinate)** |
| GNAS_fwd | GCTTCCTGGACAAGATCGAC | chr20:57484420 chr20:57484596 |
| GNAS_rev | CTCCACAAACCTGTTGTTCCA |  |
| GNAS_short_fwd | GCCGAGCGATCAGGTGTG |  |
| GNAS_short_rev | GGAAGTTGACTTTGTCCACC |  |
| BCHE_1_fwd_1 | TCATCTTTATTAACACCCACCAAA | chr3:165547863 |
| BCHE_1_fwd_2 | TGAAGCATTTGTTGTCCCCTA |  |
| BCHE_1_rev_1 | TGAAGCATTTGTTGTCCCCTA |  |
| BCHE_1_rev_2 | CTGAATGAAGCATTTGTTGTCC |  |
| BCHE_2_fwd_1 | TTGCCTTGATCTAAAGGAAAAT | chr3:165491266 |
| BCHE_2_rev_1 | TGATGAAGCAGAATGGGAGT |  |
| UNC5B_1_fwd_1 | CTGTCGGACACTGCCAACTA | chr10:73046626 |
| UNC5B_1_rev_1 | CTGCATCCTCCCTACCAATC |  |
| UNC5B_2_fwd_1 | CCTCTGTGCCTCCTGACCT | chr10:73051306 |
| UNC5B_2_rev_1 | TGCTGGAGCTGTAGACCTTG |  |
| DARS_1_fwd_1 | CATGTAACTCAACGTCTTGCTG | chr2:136701012 |
| DARS_1_rev_1 | GGAGTTCTGACACCTCTGATGA |  |
| DARS_2_fwd_1 | TCATCTCCCATTTCGACTCC | chr2:136673874 |
| DARS_2_rev_1 | TCAGACTGAAATTCAAACAGTGAA |  |
| DARS_2_rev_2 | AAATCATTCTGCAAATAGGTTTCA |  |
| DARS_3_fwd | CAACTCTGGGGAGAGCAAAT | chr2:136681989 |
| DARS_3_rev | GCAGTCTTCCGTCTCCAGTC |  |
| NGDN_1_fwd | CAGGGTCTCAGCTTCTTGGA | chr14:23944471 |
| NGDN_1_rev | AAGCCAAATGCTTCATACCG |  |
| NGDN_2_fwd | GGAAGTGAAAGACCAGCTGC | chr14:23944783 |
| NGDN_2_rev | TCATATTGCTGGGATGAGGCT |  |


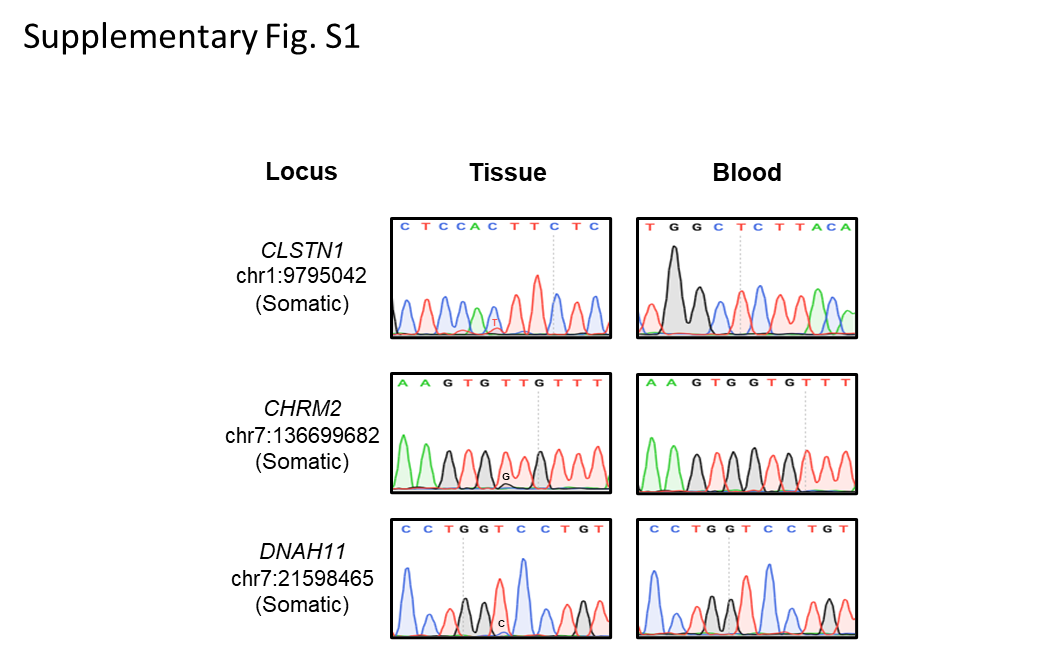


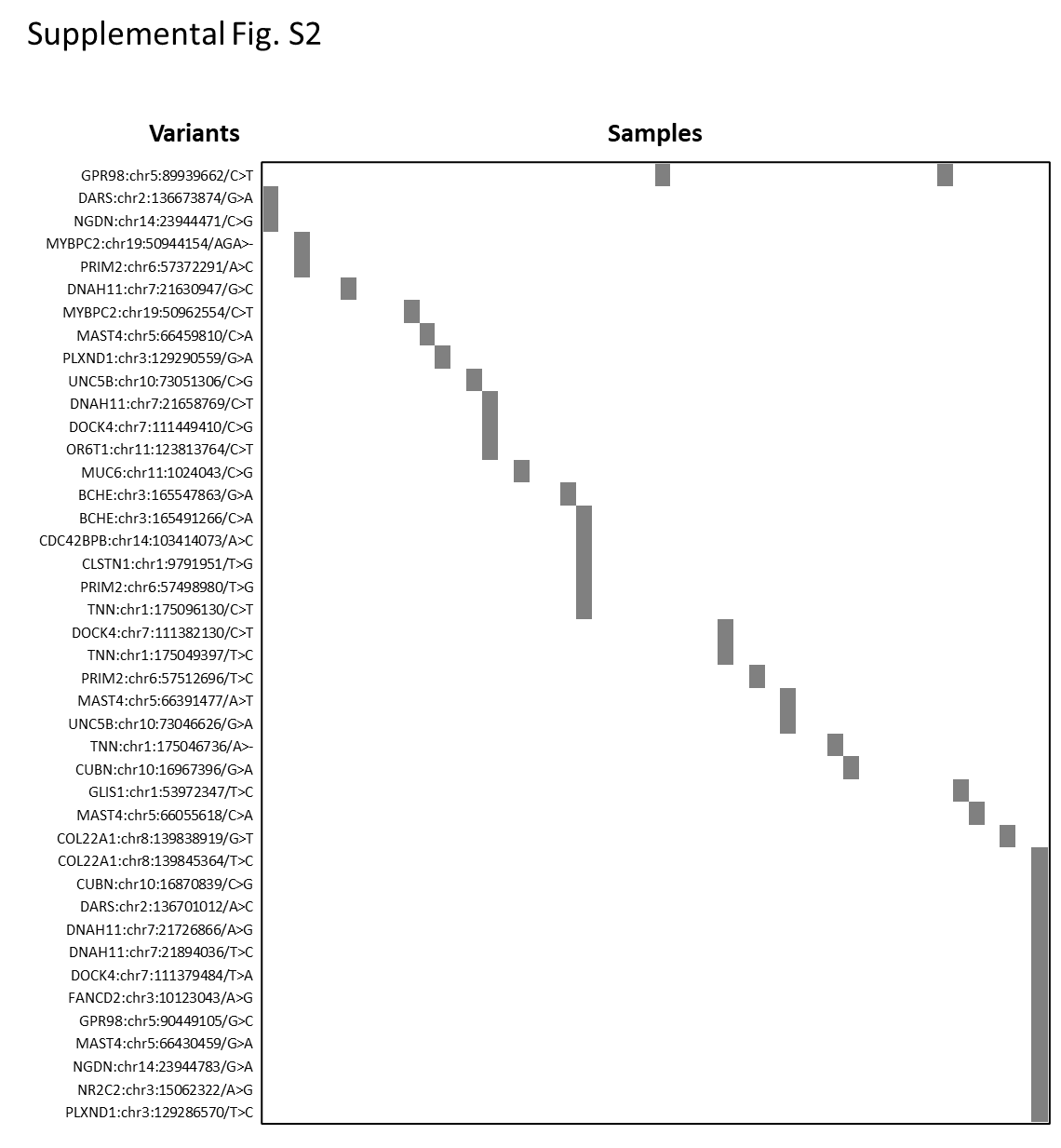


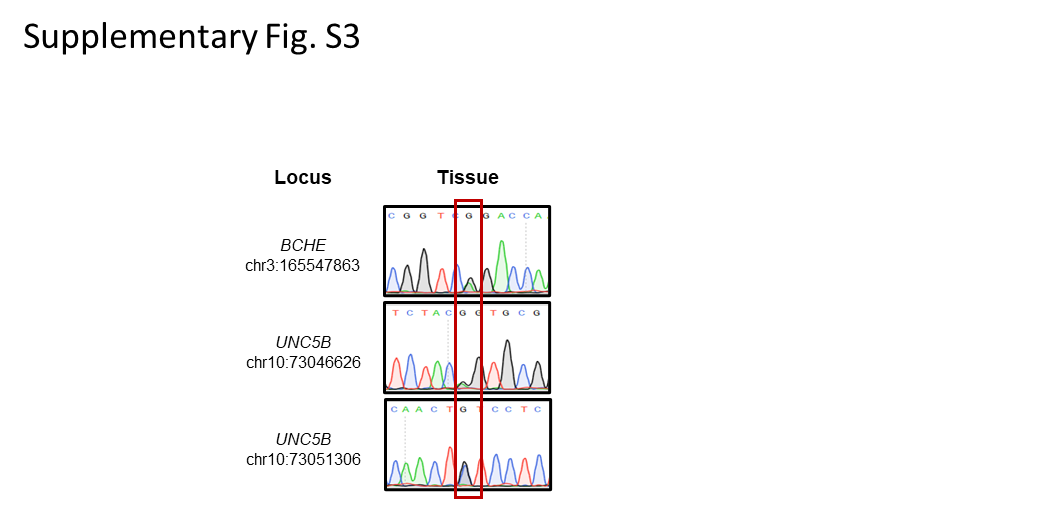


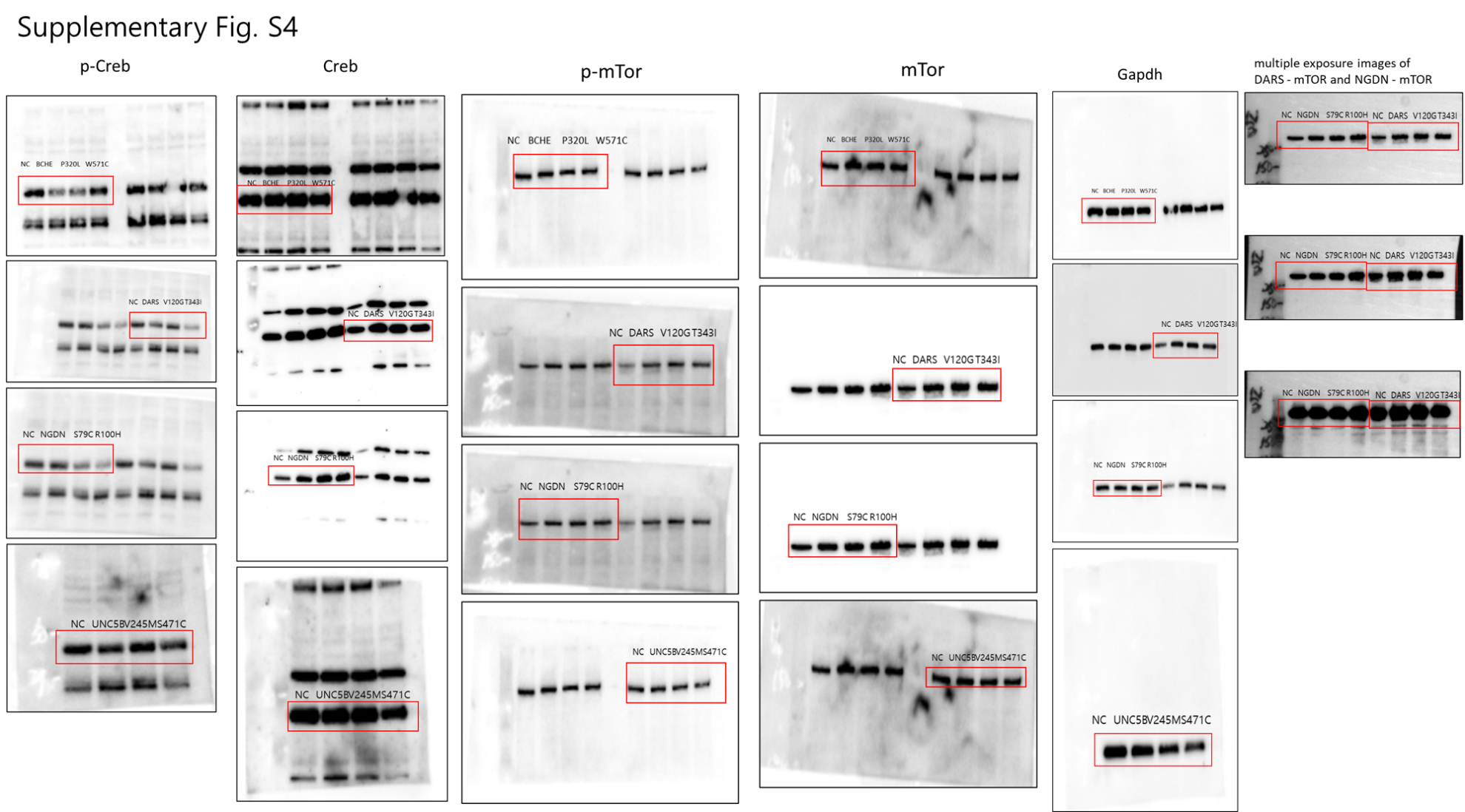


**Supplementary Figure Legends**

**Supplementary Fig. S1** Sanger sequencing results of mutations found by whole-exome sequencing (WES) (created in Snap Gene Viewer).

**Supplementary Fig. S2** Variant profiles of validation cohort. The profile was re-organized by the exact position of variant from Fig. 3b (created in Microsoft 365 Powerpoint, Excel).

**Supplementary Fig. S3** Representative images of Sanger sequencing of *BCHE* and *UNCB5*

Sequencing of the blood samples was not performed for the cohort in which only the FFPE tissue sample was available (created in Snap Gene Viewer).

**Supplementary Fig. S4** Full-length blots in Figure 4A (drawn in Microsoft 365 Powerpoint).
